# Structural equation modeling reveals determinants of fitness in a cooperatively breeding bird

**DOI:** 10.1093/beheco/arab135

**Published:** 2021-12-24

**Authors:** Michela Busana, Franz J Weissing, Martijn Hammers, Joke Bakker, Hannah L Dugdale, Sara Raj Pant, David S Richardson, Terrence A Burke, Jan Komdeur

**Affiliations:** Groningen Institute for Evolutionary Life Sciences, University of Groningen, 9700 CC Groningen, The Netherlands; Groningen Institute for Evolutionary Life Sciences, University of Groningen, 9700 CC Groningen, The Netherlands; Netherlands Institute for Advanced Study, 1012 CG Amsterdam, The Netherlands; Groningen Institute for Evolutionary Life Sciences, University of Groningen, 9700 CC Groningen, The Netherlands; Aeres University of Applied Sciences, 1325 WB Almere, The Netherlands; Groningen Institute for Evolutionary Life Sciences, University of Groningen, 9700 CC Groningen, The Netherlands; Groningen Institute for Evolutionary Life Sciences, University of Groningen, 9700 CC Groningen, The Netherlands; School of Biology, The Faculty of Biological Sciences, University of Leeds, Leeds LS2 9JT, UK; Groningen Institute for Evolutionary Life Sciences, University of Groningen, 9700 CC Groningen, The Netherlands; School of Biological Sciences, University of East-Anglia, Norwich NR4 7TJ, UK; School of Biological Sciences, University of East-Anglia, Norwich NR4 7TJ, UK; Nature Seychelles, PO Box 1310, Roche Caiman, Mahé, Republic of Seychelles; Department of Animal and Plant Sciences, University of Sheffield S10 2TN, Sheffield, UK; Groningen Institute for Evolutionary Life Sciences, University of Groningen, 9700 CC Groningen, The Netherlands

**Keywords:** cooperative breeding, extra-group paternity, reproductive output, sex differences, Seychelles warblers, structural equation model

## Abstract

Even in well-studied organisms, it is often challenging to uncover the social and environmental determinants of fitness. Typically, fitness is determined by a variety of factors that act in concert, thus forming complex networks of causal relationships. Moreover, even strong correlations between social and environmental conditions and fitness components may not be indicative of direct causal links, as the measured variables may be driven by unmeasured (or unmeasurable) causal factors. Standard statistical approaches, like multiple regression analyses, are not suited for disentangling such complex causal relationships. Here, we apply structural equation modeling (SEM), a technique that is specifically designed to reveal causal relationships between variables, and which also allows to include hypothetical causal factors. Therefore, SEM seems ideally suited for comparing alternative hypotheses on how fitness differences arise from differences in social and environmental factors. We apply SEM to a rich data set collected in a long-term study on the Seychelles warbler (*Acrocephalus sechellensis*), a bird species with facultatively cooperative breeding and a high rate of extra-group paternity. Our analysis reveals that the presence of helpers has a positive effect on the reproductive output of both female and male breeders. In contrast, per capita food availability does not affect reproductive output. Our analysis does not confirm earlier suggestions on other species that the presence of helpers has a negative effect on the reproductive output of male breeders. As such, both female and male breeders should tolerate helpers in their territories, irrespective of food availability.

## Teaser

In cooperatively breeding birds, non-parental individuals help the breeding pair with raising the young. As helpers can also have negative effects, it is not clear whether and when the breeding pair should tolerate them. We show that a statistical method (causal analysis) can shed new light on this issue. Analyzing data on Seychelles warblers, we show that reproductive success is not affected by food availability, but that both breeders profit from the presence of helpers.

## Introduction

Individuals interact with each other and with their environment, creating complex interplays that often drive fitness variations and, thus, these interplays may contribute to how many offspring are left in the next generation. Identifying the determinants of fitness and their interactions in natural populations is of fundamental importance for a wide range of applications that include, among others, animal ecology, evolutionary ecology, life history, and biodiversity conservation and management. In most animal species, the sexes differ considerably in their reproductive strategies. This is even the case in socially monogamous species without pronounced differences in secondary sexual characters. In socially monogamous groups, the breeding female and male interact with contact calls, and the male mate guards the female. Despite the mate-guarding, there is often a certain level of promiscuity (Koenig and Dickinson 2004; Taborsky et al. 2008). Males often seek reproduction outside of the group freeing them from providing parental care to those offspring. In contrast, females might seek extra-group mating to increase the genetic diversity of the offspring. For example, in cooperative breeders, males compete with each other to obtain extra-group matings, and some individuals are more successful than others. Some females are also more likely to accept extra-group mating (Gerlach et al. 2012). In addition to between-individual variation in reproduction, individuals often change tactics throughout their lives, and this might be related to their intrinsic state or their social or physical environment (West-Eberhard 2003). Many drivers at the individual state and at the group level, such as the group composition or territory quality, influence reproductive output between females and males (Green et al. 1995; Brooker and Rowley 1995; Potticary et al. 2016).

In cooperatively breeding species, individuals live in socially structured groups consisting of a breeding pair and additional sexually mature birds. The breeding pair exhibits bonding behavior and can monopolize reproduction within the group (Hodge et al. 2008). Additional individuals group with the breeding pair and may or may not help the breeding pair in rearing offspring that are not their own (Koenig and Dickinson 2004; Koenig and Dickinson 2016). The presence of helpers may benefit the breeding pair’s reproductive output, although the benefits are often difficult to detect (Downing et al. 2020). The effect of helpers on reproductive output is often complicated. The presence of helpers may increase the survival of the brood produced by the breeding pair (e.g., Heinsohn 1991; Cockburn 1998; Woxvold and Magrath 2005), but the presence of several helpers may also result in increased competition for food resources reducing reproductive output of the breeding pair (Brouwer et al. 2009). Hence, the social environment (group size and number of helpers) can be a significant determinant of reproductive output of the breeding pair. Group size and the number of helpers are equivalent in species with obligate cooperative breeding. However, group size might be uncorrelated with the number of helpers in species with facultatively cooperative breeding and facultative helping. The term facultative implies that the breeding pair might reproduce with helpers, or with individuals that live in the same group but do not provide any help, or with a mixture of individuals that help and do not help, or in the absence of other individuals (Koenig and Dickinson 2004; Komdeur et al. 2016). Therefore, in species where not all group members participate in raising the brood, it is necessary to distinguish between group size and number of helpers and test for their effect simultaneously. These species represent a unique opportunity to tease apart the effect of group size and helpers on reproductive output of female and male breeders.

Another fundamental characteristic of cooperative breeding species is group territoriality (Gaston 1978). Groups defend territories of defined shape and size, and rely on the territory for food and protection for the offspring. Territory quality has also been shown to be a good predictor of reproductive output (Koenig 1981; Komdeur 1992). However, territory quality is an abstract concept that refers to any characteristics of the territory that increase the fitness of individuals, and their causal link to reproduction might be unclear (Ens et al. 1992). The essential characteristics of the territory that enhance reproductive output differ between species, but larger territory sizes and better food availability are likely to be a positive determinant of reproductive output (e.g., Weatherhead and Robertson 1977; Stacey and Koenig 1984; Both and Visser 2000). In promiscuous species, males from larger territories are more likely to sire offspring outside the social group, but it might also be harder for them to prevent their partners from gaining extra-group offspring (Brooker and Rowley 1995). Not only territory size, but also food abundance present in the residence territory may be associated with rate of extra-group paternity (EGP) and cuckoldry of the male territory owner. In socially monogamous species, EGP is the result of fertilization by a male outside the social group. The number of extra-group offspring produced might be higher both under low food availability (females might seek better breeding opportunities outside the residence territory, Charmantier and Blondel 2003; Rubenstein 2007) or high food availability (females might be more effective at resisting mate guarding and produce more offspring extra-group, Hoi-Leitner et al. 1999). Therefore, territory quality might differently affect reproductive output of females and males.

Especially in long-lived species, such as most cooperatively breeding birds, age is a strong predictor of reproductive output (Downing et al. 2015). Reproductive output generally increases with age but declines later in life because of a physiological degeneration or senescence (Hammers et al. 2019, 2015). Typically, senescence patterns in reproductive output differ between the sexes (Bonduriansky et al. 2008; Beirne et al. 2015). However, in some species, females and males senescence at similar rates (e.g. Seychelles warblers show a decline in reproductive output after six years of age in both females and males, Komdeur 1996). Therefore, it is important to examine the combined effects of age, the social environment, and territory quality on the reproductive output of female and male breeders. In facultatively cooperative breeders, the expectation is that all three factors have an effect and that the presence of helpers affects the reproductive output of both females and males.

The Seychelles warbler (*Acrocephalus sechellensis*) is an excellent species to investigate the relative importance of the social and environmental factors on breeding success. Seychelles warblers are promiscuous passerines with facultatively cooperative breeding (Komdeur et al. 2016). Individuals live as pairs or in groups comprising a breeding pair and a variable number of individuals, of which some provide care for the offspring (helpers), while others do not (nonhelpers). About 40% of the offspring are produced extra-group (Richardson et al. 2001). Males additional to the breeding pair very rarely reproduce (2.5% of offspring were produced by additional males, Raj Pant et al. 2019). Intra-specific nest parasitism by females across groups does not occur (Richardson et al. 2001; Hadfield et al. 2006). Female helpers can become co-breeders by laying an egg in the nest together with the dominant female, and breeding males can gain paternity by copulating with helper females (11% of offspring are produced with helper females, Raj Pant et al. 2019) both within- and extra-group (Richardson et al. 2001; Raj Pant et al. 2019). The species has been intensively studied, and accurate measures of age, sex, parentage, social status (breeder, helper, nonhelper), and territory quality have been measured from 1995 onwards.

The analysis of potential drivers of fitness components has been traditionally carried out using multiple regression analysis with multilevel effects to account for individual and social group quality and correct for pseudoreplication in the data structure. However, multiple regression analysis is not able to disentangle complex relationships between variables. For example, even when a correlation between a driver and a fitness component is found, there could be a confounding variable driving variations in both variables. Structural equation modeling (SEM; Shipley 2002; Hoyle 2012) represents a better alternative because it infers cause-effect processes in complex systems through the addition of a structural model (Grace et al. 2012). SEM combines statistical principles with pre-existing knowledge to test hypothetical causal dependencies between the variables of interest (Lee and Song 2012). These dependencies can be represented as graphical path models and often include nonlinear effects between variables (Grace et al. 2012). Importantly, SEM estimates the likelihood of the different graphical path models taken into consideration. The comparison of the likelihoods tells us which path is best supported by the data. As such, SEM does not provide evidence of causality strictly, but it instead promotes the investigation of causal dependencies. SEM is particularly useful when it is not possible to use randomized manipulative experiments to disentangle causal dependencies (Grace et al. 2012). The utility of SEM in answering ecological and evolutionary questions is slowly starting to be recognized, but it is not yet widely applied (but see Fan et al. 2016). Here we apply SEM to investigate the relative importance of individual, and social and physical environmental traits for breeding success, using the facultatively cooperatively breeding Seychelles warbler.

In a facultatively cooperative breeding system with a high level of EGP, the social environment and territory quality might differentially affect the reproductive output of male and female breeders. For example, in some cooperatively breeding species only female breeders profit from helping while male breeders often lose paternity in the presence of helping because of a higher level of cuckoldry (Varian-Ramos et al. 2012; Potticary et al. 2016). In Seychelles warblers, the probability that a breeding male is cuckolded is positively related to group size and not to the number of helpers (Raj Pant et al. 2019). The aim of this study is to investigate whether females and males differ in reproductive output in response to variation in social environment and territory quality, correcting for differences in age of breeders. We formulated ten alternative structural equation models, each corresponding to a different hypothesis on the causal pathways underlying differences in reproductive output. The SEM method first calculates the likelihood of the set of field data given each of these models and subsequently allows to single out the models that are best supported by the data. In the most complex model, all variables influencing reproductive output (namely the social environment, territory quality, and age) were allowed to vary between breeding females and males. Progressively, the most complex model was simplified by setting some parameters equal between breeding females and males. The evaluation of the likelihood of the models is an essential process to assess competing pathways. The comparison of the likelihoods informs us if the causal relationship between the variables of interest and reproductive output varied between breeding females and males. Finally, we formally compared conclusions from the SEM analyses with previous studies using generalized linear (mixed) models (GLM/GLMM). To do so, we ran additional GLM/GLMM on our dataset to replicate the findings of Komdeur (1992) and Brouwer et al. (2009), who analyzed the reproductive output of Seychelles warblers in different years.

## MATERIALS AND METHODS

### Study species and data collection

We collected data on Cousin Island (Seychelles,$29\;km^{2}$,$4^{\circ }20^{\prime }\;S$, $55^{\circ }40^{\prime }\;E$) from 1995 to 2016. The population was at a carrying capacity of circa 320 individuals occupying circa 110 territories and there is virtually no dispersal to other islands (Komdeur et al. 2016). Seychelles warblers were captured using mist-nets and were ringed with unique combinations of color rings and a metal BTO. The majority of birds were captured in their first year of age, and a mixture of behavioral and morphological characteristics was used to estimate their age in years (Richardson and Burke 2003). A blood sample (circa 25μl) was collected by brachial venipuncture for sexing and DNA parentage analyses (Richardson et al. 2001; Hadfield et al. 2006; Edwards et al. 2016). Parentage was assigned using 30 highly polymorphic microsatellite loci and with a probability equal or higher than 80% (Richardson et al. 2001; Sparks et al. 2021). Circa 97% of the population, including the young birds, was individually ringed and sampled (Komdeur et al. 2016). These unique features of the population enabled us to describe accurate life histories of the individuals in the population (Raj Pant et al. 2019) and determine accurate parentage (Sparks et al. 2021).

Individuals live in year-round territories and groups range from 2 to 8 individuals of at least 6-months of age. During the main breeding season (May–September), all territories were checked to determine the presence and status of individuals. In each territory, individuals were classified as breeding females and males if they displayed pair-bonding behaviors (Richardson and Burke 2003). The behavior of the additional individuals of at least six months or older was observed closely; some of these individuals were observed incubating and or feeding offspring, and therefore classified as helpers. Female helpers were also sometimes co-breeders laying an egg in the same nest of the breeding pair (11% of offspring were produced by helper females, Raj Pant et al. 2019). The reproductive output of helpers (including co-breeders) and nonhelpers was not analyzed here. For nests that failed during the nest-building stage, it was not possible to classify additional individuals as helpers or not. For those nests we assumed that an individual that helped both in the previous and following years would have helped had the nest been successful. The number of individuals additional to the breeding pair and to the helpers determined the size of the group. Territory boundaries were determined by observing territorial disputes with neighboring birds. Territory size was calculated using the ArcGIS 10.2 (ESRI) software. Given that Seychelles warblers exclusively feed on insects from the underside of leaves (Brouwer et al. 2006; Komdeur et al. 2016), insect food availability in a territory was estimated each month during the main breeding season as the number of insects present and vegetation cover per territory (see Supplementary Appendix A8 for further details and Komdeur 1992). The combined measures of territory size and food availability are good proxies to estimate the quality of each territory (Komdeur 1992; Brouwer et al. 2006).

**Figure 1:**
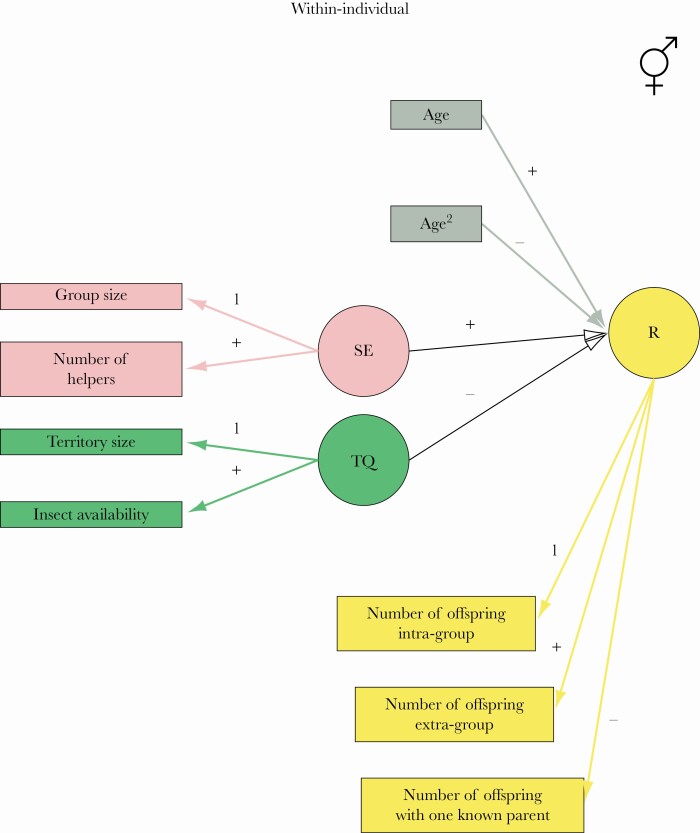
The path diagram is representing the causal relationship within-individual in the two-sample three-level structural equation models (SEMs) M1 to M6. Circles represent the latent variables (R = reproduction potential, SE = social environment, and TQ = territory quality), while rectangles represent fixed covariates and observed variables. To highlight the relationship between observed and latent variables, we colored variables related to the social environment in pink, to territory quality in green, to reproduction in yellow. Fixed covariates (age, age^2^) are colored in grey. The path represents both the female and male samples. Black lines represent causal effects on reproduction, while the pink, green, and yellow lines represent the manifestation of the latent variables into observed variables. Grey lines represent the correlation between reproduction and fixed covariates. These relationships are present in all the competing models, but some of these lines are kept invariant between females and males in different models. The plus and minus signs at the top of each line indicate if the parameter is positive or negative. The thickness of the lines is proportional to the relative importance of the corresponding parameter estimate in M6, where all the lines are invariant between females and males.

Over the 21 years of data collection, 419 breeding females and 442 breeding males were observed for a minimum of two years for a total of 2271 and 2229 reproductive attempts, respectively. Yearly reproductive output of a breeder was defined as the number of genetic offspring produced in a given year that survived to at least one year of age. Offspring were classified as within-group (n=439) if the genetic parents were also socially linked, or extra-group (n=317) when parents belonged to different territories. For 107 offspring we determined the identity of the mother but not of the father and for 150 offspring the identity of the father but not of the mother. The individual-level variables measured (the number of offspring produced by the breeder within- and extra-group, the number of offspring with only one known parent, and age) and group-level variables (group size, the number of helpers in a territory, territory size, and insect availability) are reported in Figure 1 and in the Supplementary Appendix (A8 and Table A1).

### Implementation of SEM

SEM is a multivariate regression technique that analyses linear and nonlinear causal relationships among a set of variables (Kline 2011). The SEM method consists of comparing various biologically plausible hypotheses on how a response variable, such as reproductive output, is caused by some measured variables, such as insect availability, and also some latent variables that cannot be directly measured (see below). For each hypothesis, a structural equation model is constructed that can be visualized by a causal pathway, where the hypothesized cause-effect relationships are represented as arrows between the variables of interest. For each of these structural equation models, the likelihood of the field data can be determined, allowing to rank the competing models (and their underlying causal hypotheses) on the basis of their empirical support. Importantly, SEM allows the inclusion of latent variables that cannot be directly measured, such as territory quality and social environment. These variables manifest attributes that are directly measured, and it is possible to use statistical methods to derive their values from associations among measured variables (Lee and Song 2012). For example, territory quality is a latent variable that can be evaluated by simultaneously measuring food availability and territory size. Latent variables circumvent errors due to collinearity and are extremely common in biology because biologists often tend to measure theoretical and complex concepts that are expressed by a multitude of traits (Mitchell 1992). From a practical point of view, within each latent variable, we choose an observed variable as the reference level (Cubaynes et al. 2012). For example, we selected the territory size as the reference variable for territory quality. Therefore, the parameter value for territory size was 1. The parameter value for insect availability indicates the relative importance of the variable compared to the reference level (Figure 1 and Supplementary Appendix A3).

Here we built two-sample (females and males) three-level SEM to estimate variation in reproductive output among females and males at the three levels of within-individual, between-individuals, and between-groups. By including the individual level in the model, we accounted for the fact that some observations are not independent but belong to the same individual. In other words, the within-individual variations inform us about how changes in the variables affect the reproductive output of an individual within its lifetime. The between-individuals level assesses characteristics of individuals that are invariant with time so that it can be interpreted as a measure of individual quality. We also included the between-groups level as a third level to correct for pseudo-replication because a female and male belong to the same social group in a given year. The three-level approach is similar to a hierarchical model within the linear regression framework. For example, for every parameter, the algorithm estimates the expected reproductive output of each individual (mean and standard variation, Lee and Song 2012). The two-samples inclusion allows testing for similarities or differences among the sexes by estimating parameter estimates for the females and males (Thanoon and Adnan 2015). The method is similar to the inclusion of a factor as an explanatory variable (e.g. sex as female and male) in linear regression analysis, where the algorithm estimates parameters for each factor level and its interactions with other explanatory variables in the model. The models were implemented in a Bayesian framework, and model parameters were estimated through Markov chain Monte Carlo (MCMC) samples (Lee and Song 2012). Model performance was evaluated by applying posterior predictive checks on the MCMC samples through the Gelman-Rubin statistics ($\hat{R}\;\leq 1.05$, Gelman and Meng 1996) and by inspecting plots of the chains and the residuals of the model (Lee and Song 2012).

The vectors $y_{♀}{}_{io}$ and $y_{♂}{}_{io}$ represent the observed variables with regards to the female (♀) and male (♂) samples respectively. The subscripts represent each observation *o* of individual *i*. The variables $y_{♀}{}_{io}$ and $y_{♂}{}_{io}$ are ordinal with three, four, or five categories. To ensure each variable was normally distributed we transformed each $y_{♀,♂io}$ into its continuous underlying variable ${y^{\prime }}_{♀,♂io}\sim N\left[0,\sigma^{2}\right]$ (Lee 2007; Gelman et al. 2014; Thanoon and Adnan 2015). The complete formulas describing the SEM and the transformation of ordinal variables by means of thresholds can be found in the Supplementary Appendix (A2–A5, Figure A1).

### Implementation

To quantify the effect of the social environment, territory quality, and age on the reproductive output of females and males, we built ten competing models. Each model includes two exogenous latent variables (social environment and territory quality) and an endogenous latent variable (reproductive output). The social environment quantifies the level of positive and negative interaction the breeding individual has with other individuals within the same groups and is measured by the group size and the number of helpers present. Territory quality expresses the variation in resource availability among different territories and is measured as the size of the territory and arthropod prey availability in respect of the vegetation cover within the territory. Age (years) is treated as a fixed covariate as reproductive senescence occurs in Seychelles warblers (i.e., reproductive output increases over early ages until a plateau is reached, followed by a decline later in life, Hammers et al. 2012). Consequently, we included a quadratic effect of age on reproduction. Yearly reproductive output expresses the ability of a breeder to pass its genes to the next generation by producing one or multiple offsprings. This was measured by the total number of offspring produced intra-group, extra-group, or with only one known parent in a given year.

To investigate possible differences among females and males while accounting for individual variation, we implemented two-sample (females and males) three-level SEMs. By setting a parameter to be invariant between female and male samples, we are building a scenario where there is no difference between females and males concerning that specific parameter. By comparing the likelihoods of different models, we can formally test if there are substantial differences between the sexes. We built ten competing models by setting some parameters to be invariant over the females and males, as described in Table 1. Since previous studies showed that the rate of reproductive senesce is similar between females and males (Komdeur 1996), in all the models the onset of senescence was invariant between sexes. With each model, we tested for differences in the causal effect of one specific variable at a time. For example, in M5 variations in the social environment, but not in territory quality, were affecting the reproductive output of females and males at a different rate. In M6 changes in the social environment and in territory quality were causing similar changes in the reproductive output of females and males. Therefore, the likelihoods of M5*versus*M6 can compare a scenario where reproductive output depends on the social environment at a different rate for males and females *versus* a scenario where females and males are similarly affected by the social environment.

**Table 1:** Ten models considering various types of potential sex differences in the effects of social environment, territory quality and age on reproductive output. In models M1 to M6 the effect of territory quality on reproduction was direct, while in models M7 to M10 we tested if the effect of territory quality on reproduction was mediated through variations in the social environment. Similarities between females (♀) and males (♂) are tested by applying constraints on different parameters. The M1 is the full model with unconstrained parameters (≠). We progressively set invariant parameters (=) in the following models. In M2 the relationship between the observed variables and the corresponding latent variable were set to be equal between the sexes. Additionally, in M3 and M7, we set the values of the latent variable to be equal between females and males when the observed variables are 0. Similar interpretations are commonly applied to the comparison of slopes and intercepts between two linear regressions lines. Finally, we tested the invariance of the latent variables. In M4 and M8, the social environment was invariant, while the territory quality was unconstrained. In M5 and M9, the social environment was unconstrained while the territory quality was invariant. In M6 and M10, both the social environment and territory quality were invariant between females and males

	Social environment			Territory quality		
Model	Effect on reproduction	Intercept of the observed variables	Slope of the observed variables	Effect on reproduction	Intercept of the observed variables	Slope of the observed variables
M1	♀ ≠ ♂	♀ ≠ ♂	♀ ≠ ♂	♀ ≠ ♂	♀ ≠ ♂	♀ ≠ ♂
M2	♀ ≠ ♂	♀ ≠ ♂	♀ = ♂	♀ ≠ ♂	♀ ≠ ♂	♀ = ♂
M3	♀ ≠ ♂	♀ = ♂	♀ = ♂	♀ ≠ ♂	♀ = ♂	♀ = ♂
M4	♀ = ♂	♀ = ♂	♀ = ♂	♀ ≠ ♂	♀ = ♂	♀ = ♂
M5	♀ ≠ ♂	♀ = ♂	♀ = ♂	♀ = ♂	♀ = ♂	♀ = ♂
M6	♀ = ♂	♀ = ♂	♀ = ♂	♀ = ♂	♀ = ♂	♀ = ♂
**Model**				Effect on social environment		
M7	♀ ≠ ♂	♀ = ♂	♀ = ♂	♀ ≠ ♂	♀ = ♂	♀ = ♂
M8	♀ = ♂	♀ = ♂	♀ = ♂	♀ ≠ ♂	♀ = ♂	♀ = ♂
M9	♀ ≠ ♂	♀ = ♂	♀ = ♂	♀ = ♂	♀ = ♂	♀ = ♂
M10	♀ = ♂	♀ = ♂	♀ = ♂	♀ = ♂	♀ = ♂	♀ = ♂

Models M7, M8, M9, and M10 represent a scenario with an indirect effect of territory quality on reproduction (Supplementary Figure A2). Territory quality causes variations in the social environment, which in turn determines the reproductive output of females and males. In model M7, the parameter values linking the latent variable are different between females and males. In model M8, the effect of social environment was allowed to vary between females and males, and the effect of territory quality was kept invariant. In model M9, the effect of the social environment was kept invariant, while the effect of territory quality could vary between the sexes. In models M10, the parameter values were invariant between females and males.

All models were estimated in JAGS 4.3.0. The packages rjags 4–10 (Plummer 2016) and doMc 1.3.7 (Revolution Analytics and Weston 2015) were used to run parallel chains within the R environment (R 4.0.0, R Core Team 2020). Additional details are provided in the Supplementary Appendix (Section A6). The variables group size, number of helpers in a group, territory size, and insect availability included missing data, which were classified as missing at random (Little and Rubin 1989).

### Model comparison

To compare the validity of the competing models, we calculated the $L_{\nu }-$ measure, which is a Bayesian criterion-based method (Ibrahim et al. 2001). The $L_{\nu }-$ measure quantifies the performance of a model M in the light of the continuous data Y in terms of two undesirable properties: the variation in the model predictions and the discrepancy between model predictions and observations. The term ν is a parameter that varies between zero and one and sets the weight of the first component compared with the second. For example, when ν=1, the two components have equal weight; when ν=0 the second component becomes zero. It is common practice to set ν=0.5 to give more weight to the variation in the model predictions (Song et al. 2011). When ν=0.8 the discrepancy between predictions and observations is given more weight. Here, we calculated the $L_{\nu }-$ measure with a ν=0.5 to follow the recommendation, and also with a ν=0.8. Given a model M and continuous data Y, for i=1,⋯,N, $o=1,\cdots ,N_{i}$, $s=1,\cdots ,N_{s}$ the $L_{\nu }-$ measure is calculated as follows:


$$\begin{array}{l} L_{\nu }(Y,M)=\underset{s=1}{\overset{N_{s}}{\sum }}\;\underset{i=1}{\overset{N}{\sum }}\;\underset{o=1}{\overset{N_{i}}{\sum }}\;Var(y_{sio}^{rep}|Y,M)+\nu \underset{s=1}{\overset{N_{s}}{\sum }}\;\underset{i=1}{\overset{N}{\sum }}\;\underset{o=1}{\overset{N_{i}}{\sum }}\;{\left[y_{sio}^{rep}-E(y_{io}^{rep}|Y,M)\right]}^{2} \end{array}$$ (1)

where $y_{sio}^{rep}$ indicates the imaginary replicates of Y for each observation o of individual i nested in a social group s. Equation 1 is a function of the values of the predicted and observed data and, as such, the absolute values of the $L_{\nu }-$ measure are highly dependent on the sample size. The relative difference between $L_{\nu }-$ values is used to compare the models, and the model with the lowest $L_{\nu }-$ value should be preferred as the best model fitting the data (Lee and Song 2012). When models have similar performance (e.g. $\Delta L_{\nu \;}<\;2$) the simplest model is chosen according to the principle of parsimony. Formulas to calculate the $L_{\nu }-$ measure for SEM with ordinal data can be found in Li and Yang (2011); Song et al. (2011).

### Comparison with previous studies

Seychelles warblers’ reproduction has been traditionally analyzed using a correlational generalized linear (mixed) model (GLM/GLMM) approach (e.g., Komdeur 1992; Brouwer et al. 2009). The SEM approach is complementary to previous studies because it investigates the causal relationships between the social environment and territory quality on reproduction. SEM compares the reproduction of females and males in response to variations in social environment and territory quality, including indirect effects that cannot be tested with GLM/GLMM. However, the comparison of SEM with previous studies is flawed. When discrepancies are found between the SEM and GLM/GLMM results, it is hard to disentangle how they arise. On the one hand, potential differences might be linked to biological changes through time, when different timeframes of data are analyzed. On the other hand, discrepancies might be methodological and linked to the different statistical approaches or definitions of variables. To better compare our results with previous studies, we run additional GLM/GLMM on the long-term dataset following the analysis from: Komdeur (1992), who analyzed the effect of territory quality and the number of helpers on reproduction; and Brouwer et al. (2009), who analyzed the effect of relative territory size and group size on reproduction. We predict that if the GLM/GLMM produce similar general conclusions to those of SEM on the same dataset but different conclusions from previous studies (which analyzed different timeframes of data), there have been biological changes through time that can explain why differences with previous studies are found. Technical details are reported in Supplementary Appendix A9.

## RESULTS

All models passed our inspection diagnostics. For example, the Gelman-Rubin statistics were <1.05, and the trace plots showed that all chains converged to the same distribution.

### Model ranking

The model M6 followed by M3 (top models) performed better than the other models in explaining the data on the reproductive output of female and male Seychelles warblers with the $L_{\nu =0.5}-$ measures (Table 2) and the $L_{\nu =0.8}-$ measures (Supplementary Appendix Table A4). We consider M6 to be the most parsimonious model because M6 contains fewer parameters and only direct effects. The models containing an indirect effect of territory quality on reproduction mediated through variations in the social environment received little support (Table 2).

**Table 2:** Model ranking of the competing models based on the *L*_*v*_ = 0.5 measure. The top-ranking model was M6 followed by M3. Models allowing for an indirect effect of territory quality on reproduction mediated through variations in the social environment (M7 to M10) received little support

Id	$L_{\nu }=0.5$
M6	21767.88
M3	21791.88
M1	21793.53
M4	21798.95
M5	21801.38
M2	21803.85
M8	22127.84
M9	22271.19
M7	22285.01
M10	22297.72

### Top model describing the influence of social environment and territory quality on reproductive success

**Figure 2:**
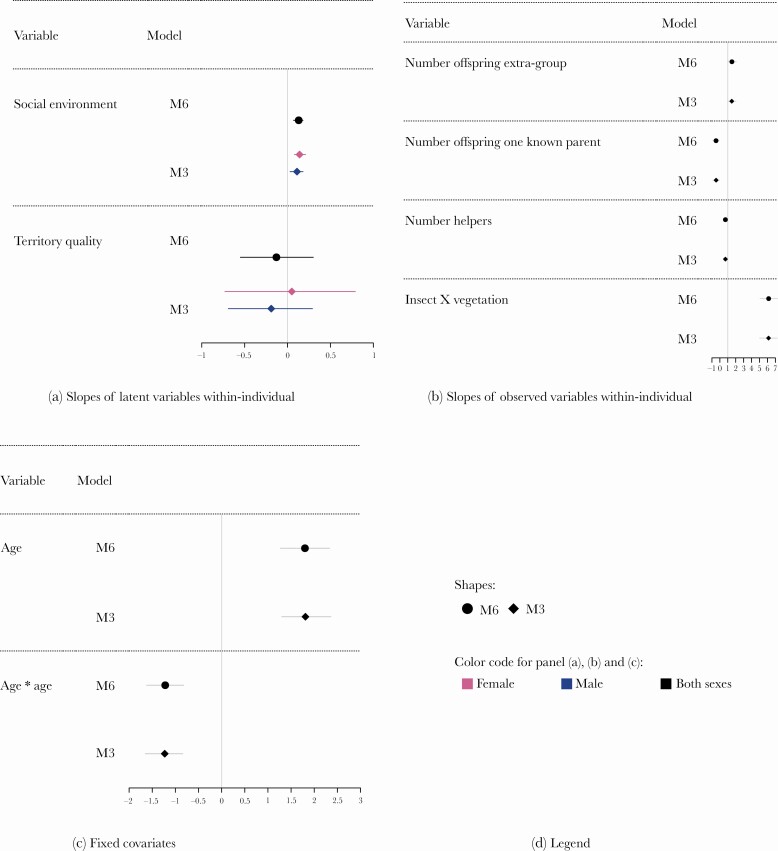
Mean of the parameters in the top two models: M6 (filled circles), and M3 (diamonds). Lines around the means represent the 95% high-posterior density intervals (HPDI) of the parameters. Four panels are representing the within-individual effect of the latent variables (a); the factor-loadings of the observed-variables (b); the values of the onset of senescence (c); and the legend of the plots (d). In Panel (a) some parameters vary between females (pink or light gray symbols) and males (blue or dark gray symbols) in M3. More specifically, in M3 both the social environment and territory quality slopes vary between females and males. However, even in M3 the 95%HPDI of the two sexes for the social environment and territory quality highly overlap, confirming that possible differences between females and males are nonsignificant (Panel (a)). The other plots represent the observed variables and the parameters are invariant between females and males (black symbols). The mean and 95%HPDI of each parameter are almost identical in the two models (Panels (b), and (c)), indicating that the two models are in good agreement. In Panel (b) the slopes of the observed variables omit the reference variable for each latent variable, namely the number of offspring intra-pair for the reproductive output, the group size for the social environment and territory size for the territory quality. These parameters are omitted here because they were set to 1 a priori for identifiability issues and to make the observed variables comparable. Therefore, the values of the parameter estimates express the importance of the observed variables to the corresponding latent variable relative to the baseline observed variable (set to be 1).

We first describe model M6 and subsequently discuss the differences between M6 and M3. According toM6, the effect of the social environment, territory quality, and age on reproductive output were similar for breeding females and males. The mean estimates of the parameters according to M6 are shown in Figure 1 and their 95% high posterior density intervals (HPDI) in Figure 2. The social environment had a significant positive effect on the variation in reproduction through the life of both female and male Seychelles warblers. Territory quality did not have an effect on reproduction within-individual (the mean parameter of the variations in reproductive output across the age of an individual was close to zero), and the large 95%HPDI overlapping zero showed some uncertainty in the estimation of the parameter (Figure 2(a)). The impact of the social environment and territory quality had no effect on between-individual differences in reproductive output for both females and males (Supplementary Appendix Figure A3(a)). The large 95%HPDI, which overlapped with zero suggest that individual quality does not affect the relationship between either social environment or territory quality and reproductive output. There were no sex differences in the onset of reproductive senescence. As individuals aged their reproductive output increased, but there was a decline after seven years of age due to senescence (Figure 2c).

It is also important to focus on the relationship between the observed variables and the latent variables. The model showed no sex differences in the slopes of the observed variables within-individual (Figure 2b) and between-individual (Supplementary Appendix Figure A3(b)). However, for the latent variable reproduction at the within-individual level, there was more variation in the number of extra-group offspring produced than of within-group offspring produced. In comparison, the production of offspring with only one known parent did not significantly influence the reproductive output within-individual (Figure 2b). This result suggests that the reproductive attempts where we could only determine a parent’s identity did not bias our overall results. The values of the slopes of the observed variables for the between-individual variation showed a different trend. The production of within-group offspring was driving variation in reproductive output between individuals (Supplementary Appendix Figure A3(b)). Within- and between-individual variation in the social environment was driven by group size and then by the number of helpers (Figure 2b, and Supplementary Appendix A3(b)). Variation in territory quality within-individual was mainly driven by insect availability rather than territory size (Figure 2b), while the effect of insect availability was minimal compared to territory size at the between-individual level (Supplementary Appendix Figure A3(b)).

In the main text, we focus on within-individual variation, while we briefly touch on between-individual and between-group effects (see for more details Supplementary Appendix, Section A7). Additional parameters values, such as error estimates and threshold estimates for the ordinal variables are also reported and discussed in the Supplementary Appendix (Section A7, Supplementary Figure A3, and Supplementary Table A3).

### Comparison between the top-ranked models

To better compare the top-ranking models, we also reported the parameter estimates and their 95% HPDI in models M3 (Figure 2). Model M3 differs from model M6 because the effects of the social environment and territory quality on reproductive output were different between females and males. The slope of the social environment was allowed to vary between the sexes in M3 but set invariant in M6. The slope for territory quality was allowed to vary between sexes in M3 and set invariant in M6. Despite these differences in the constraints imposed on the two models, the parameter estimates were very similar, both for the two sexes and across models (Figure 2a, and Supplementary Appendix A3(a)). In fact, the 95% HPDI for the parameter values were overlapping considerably.

### Results GLM/GLMM

The GLM/GLMM analysis replicated the statistical analyses presented by Komdeur (1992) and Brouwer et al. (2009) on the long-term dataset collected between 1995 and 2016. The GLM confirms Komdeur (1992)’s findings of a positive effect of the number of helpers, but in contrast with Komdeur (1992), we found no effect of territory quality on reproduction. The GLMM confirms Brouwer et al. (2009)’s finding of a positive effect of group size on reproduction, but here we found no effect of territory size. Additional results are reported in Supplementary Appendix A9.

## Discussion

We investigated the effect of EGP and group-level traits (both within and extra-group offspring) on the reproductive output of females and males Seychelles warbler, a bird species with facultatively cooperative breeding. When using SEM, overall, there were no sex differences in the reproductive output of Seychelles warbler breeders in relation to group-level traits. We found that the social environment and territory quality effect on variance in reproductive output was similar for breeding females and males. There was a positive effect of the social environment on within-individual reproductive output. Both females and males produced more offspring in the presence of larger groups and helpers. In contrast, territory quality had no effect on reproductive output in both sexes. These associations suggest that overall, there are no sex differences in the reproductive output of Seychelles warblers in relation to group-level traits when analyzed with SEM.

In strictly genetically monogamous species, the reproductive output of a breeding female and male of the same pair or group are identical in a given reproductive season. However, in promiscuous species, males can lose paternity within their own female and/or gain paternity outside their group, while females are restricted to the number of offspring they themselves produce in the group. Consequently, variability in the number of offspring produced by males and female breeders belonging to the same group can be large. Both females and males can gain benefits from extra-group mating. Females might seek to increase the genetic diversity of the offspring or search for new partners in the eventuality of mate loss (Petrie and Kempenaers 1998). In contrast, the main benefit to males will be to produce (extra) offspring without incurring the cost of parental investment (Westneat and Stewart 2003). However, males will pay costs when they themselves are cuckolded. In cooperative breeding species, the presence of helpers may free both female and male breeders from some of the costs of parental care (Koenig and Dickinson 2004), but can also increase the risk of cuckoldry for males (Green et al. 1995). Territory quality might also be negatively (Charmantier and Blondel 2003) or positively (Hoi-Leitner et al. 1999) related to the chances of losing or gaining EGP (Raj Pant et al. 2019). Given that infidelity is associated with a high level of cuckoldry in many birds species (Green et al. 1995; Varian-Ramos et al. 2012; Potticary et al. 2016), one could expect positive effects of the social environment on the reproductive output of breeding females, but adverse or null effects on males and possible different effect of territory quality on females and males. In the Seychelles warbler, there is evidence that group size promotes the likelihood of within-group paternity loss by breeding males (Raj Pant et al. 2019). Overall, our results suggest that females and males gain more reproductive output when living in a large group and groups with more helpers, but that territory quality does not affect their reproductive output. The parameter estimates that vary between females and males in the top models have overlapping HPDI which suggests that potential differences between females and males are minimal. In the Seychelles warblers, both breeding females and female helpers can reproduce with a breeding male from a different group, but almost only breeding males obtain EGP (i.e., only 1.6% of the offspring were fathered by helper or nonhelper males from another group, Raj Pant et al. 2019). Therefore breeding males could compensate for the risk of cuckoldry by mating with female helpers from the same or a different group, but breeding females could only reproduce with a breeding male.

### The effect of social environment

There was no difference between females and males in how the reproductive output changed in response to variation in the social environment. This lack of response demonstrates that the presence of helpers and additional group members were of benefit for both females and males, because the reproductive output increased with the social environment. This benefit was also apparent when considering both the number of within- and extra-group offspring produced, which increased with the social environment. Our results partly contrast with studies in other species, i.e., the red-backed fairywren (*Malurus melanocephalus*) and superb fairywren (*Malurus cyaneus*, Green et al. 1995; Varian-Ramos et al. 2012; Potticary et al. 2016). In these species, males are cuckolded more in the presence of helpers and partly compensate by reducing their parental care while seeking EGP elsewhere (Green et al. 1995). The trade-off between gaining within-group or extra-group young might explain that there is no overall reproductive benefit to the male, such that it might seem puzzling why breeding males tolerate helpers in these species (Varian-Ramos et al. 2012; Potticary et al. 2016). A fundamental difference with the Seychelles warbler is that helpers of the red-backed and superb fairywrens do not reproduce (Varian-Ramos et al. 2012; Potticary et al. 2016). Therefore, males cannot compensate for the costs of cuckoldry by mating with helper females. Furthermore, superb fairywrens have a rate of circa 76% of extra-pair copulation (Mulder et al. 1994), which likely has co-evolved with complex trade-offs between parental care and mating behavior (Green et al. 1995). The rate of EGP in the Seychelles warbler is circa 40% (Richardson et al. 2001) and might have evolved due to different selection pressure in breeding density, inbreeding, and social system (Petrie and Kempenaers 1998; Westneat and Stewart 2003). Indeed, in the Seychelles warblers, there is no evidence for a trade-off between within-group and extra-group reproduction (Raj Pant et al. 2020).

Here, the social environment was also analyzed in terms of group size. We found no local density-dependence effects on group size, reducing the reproductive output in larger groups. This result is in contrast with previous studies on the Seychelles warbler that showed a reduced reproductive output for larger groups (Brouwer et al. 2009). However, Brouwer et al. (2009) estimated the reproductive output as the number of fledglings (offspring surviving to at least three weeks of age), while here we used the number of offsprings surviving to at least twelve months of age. This difference affects the power of statistical analyses. Because mortality is very high (39%, Brouwer et al. 2006) in the first few months of life, only a few offspring survived to at least twelve months of age. Using the number of offspring surviving up to twelve months of age increased the chances of collecting a blood sample to assign the paternity and hence may result in more complete data (fewer individuals of unknown parentage). On the other hand, our database does not include some offspring that might have died between fledgling and twelve months.

### The effect of territory quality

The second latent variable analyzed was territory quality. The SEM analysis did not support an indirect effect on reproduction of territory quality, mediated through variations in the social environment. Territory quality, which varied mainly due to variation in insect availability, and not to variation in territory size, had also no apparent direct effect on the reproductive output of females and males. The result is in contrast with previous studies on Seychelles warblers that showed that breeders living in high-quality territories had higher reproductive output than breeders living in low-quality territories (Komdeur 1992; Brouwer et al. 2009). The discrepancy is likely due to radical spatiotemporal changes in the vegetation and insect abundance on Cousin Island. Our conclusion is also supported by the GLM/GLMM analysis results that confirm there was no apparent effect of territory quality on reproduction when analyzing the long-term dataset. A conservation project started in 1986 replaced a coconut plantation with the native plants increasing quality over time but also homogenizing the variation in quality between territories (Komdeur and Pels 2005). Moreover, the confidence intervals of the parameter estimate for territory quality in our SEM were large, which suggests that there might be some hidden variation in the data that the models were not able to disentangle. Future studies should investigate spatial patterns in the environment to grasp this uncertainty. For example, the rate of extra-group mating in blue tits (*Cyanistes caeruleus*) is highly correlated with the distance to mating opportunities (Schlicht, Valcu, and Kempenaers 2015). We ignored the spatial component in our analysis. However, the inclusion of spatial autocorrelation of the data might highlight a complex correlation between reproduction, promiscuity, and the spatial structure of the environment. Finally, insect availability is measured as an annual average, but it might be that undetected temporal variation in food availability drives the rate of promiscuity and reproduction. To test this, it is necessary to run a time series analysis on the data.

### Onset of senescence

To account for individual-level variation in reproductive output, we included age as a fixed covariate in the models. In both sexes, the reproductive output initially increases with age, and it is followed by a decline later in life, suggesting that females and males invest more energy in reproduction early in their life. This finding confirms Komdeur (1996)’s previous study, where he examined a decline in reproductive output after six years of age in both female and males Seychelles warblers.

### Validity of the results

SEM compares causal relationships between competing models drawn based on previous knowledge on a system. As such, our results depend on the models considered and should be interpreted under this assumption. It is generally not possible to consider or include all potential causal relationships between the variables of interest. The number of models to be computed would be so large that computing time would be unfeasible. Moreover, some models might include causal links that are not biologically sensible or meaningful. We analyzed only models that would be biologically relevant and also considered additional less likely models that are not presented here because of space limitations. For example, we tested for sex differences in senescence patterns, but these models received little support and are not presented here.

We choose the $L_{\nu }$-measure to rank models because of its versatility and the ease to include ordinal data. There is some ambiguity in this information criterion because the values of ν were set to be 0.5 or 0.8. However, the parameter estimates in the two top-ranked models are highly consistent. This similarity confirms that the two best models are comparable and that there are no substantial differences in the reproductive output of females and males.

Finally, we recognize that there are multiple multivariate regression techniques for applying causal inference. Alternatives to classical SEM include path analysis (e.g., Shipley 1997) and piecewise-SEM (Lefcheck 2016). Each method presents advantages and disadvantages (e.g., Hoyle 2012; Hancock and Mueller 2013). We chose to implement SEM within a Bayesian framework because we aimed to test hypotheses about female and male differential responses, building on previous knowledge of the system. In addition, SEM has the advantage of allowing the inclusion of latent variables that account for measurement error and within the Bayesian framework can deal with missing values and pseudo-replication in the data structure (e.g., Rabe-Hesketh et al. 2007; Lee and Song 2012).

### Conclusions and recommendations for future research

We focused our analysis on reproductive output, which is a standard proxy for fitness. However, sex differences in response to the social environment and territory quality might arise in the survival probability of females and males. For example, Paquet et al. (2015) found that the presence of helpers decreases the survival probability of males while increasing the survival probability of females in the sociable weaver. It would be interesting to further investigate the role of the social environment and territory quality on the survival probability of female and male Seychelles warblers.

Our analyses included 21 years of data, and we were able to estimate the causal impact of the social environment and territory quality on variation in reproductive output both at the within- and between-individual levels. Different patterns arose when considering the within- versus between-individual variation confirming the importance of cross-sectional studies to understand life-history traits of long-lived species (Nussey et al. 2008).

In conclusion, we showed that despite female and male Seychelles warblers potentially gain different advantages and pay different costs due to extra-group mating, overall, there was no difference in their response to the social environment and territory quality. More specifically, the presence of helpers was associated with higher reproductive output in both females and males. This positive association is not obvious and, in contrast with what found in cooperative breeding species of the *Malurus* genera (Varian-Ramos et al. 2012; Potticary et al. 2016). Our results suggest that there are substantial differences amongst species, and there is a need to study further these relationships in other promiscuous species with or without cooperative breeding.

## Supplementary Material

arab135_suppl_Supplementary_Appendix

## Data Availability

Analyses reported in this article can be reproduced using the data and software provided by Busana et al. (2021). Code is also available on GitLab https://gitlab.com/michebio/code_sem_reveals_determinants_of_fitness_in_cooperatively_breeding_bird
