# Supplementary material for: Structural equation modeling reveals determinants of fitness in a cooperatively breeding bird
Source: Behav Ecol. 2021 Dec 24;33(2):352–63. doi: 10.1093/beheco/arab135 (PMC9015215; doi:10.1093/beheco/arab135)
Supplement: arab135_suppl_Supplementary_Appendix [file arab135_suppl_supplementary_appendix.pdf]

Appendix to "Structural equation modeling reveals determinants of fitness in a cooperatively breeding bird".

Michela Busana[*a], Franz J Weissing[a,b], Martijn Hammers[a,c], Joke Bakker[a], Hannah L Dugdale[a,d], Sara Raj Pant[a,e], David S Richardson[e,f], Terrence A Burke[g] and Jan Komdeur[a]

[a]Groningen Institute for Evolutionary Life Sciences, University of Groningen, Groningen, The Netherlands

[b]Netherlands Institute for Advanced Study, Amsterdam, The Netherlands

[c]Aeres University of Applied Sciences, Almere, The Netherlands

[d]The Faculty of Biological Sciences, University of Leeds, Leeds, United Kingdom

[e]School of Biological Sciences, University of East-Anglia, United Kingdom

[f]Nature Seychelles, PO Box 1310, Roche Caiman, Mahè, Republic of Seychelles

[g]Department of Animal and Plant Sciences, University of Sheffield, Sheffield, United Kingdom

*Address correspondence to M Busana. E-mail: m.busana@rug.nl

CONTENTS

Contents **1**

[*]m.busana@rug.nl

List of Figures 2

List of Tables 2

A1 Additional information about the variables considered 4

A2 Calculation of thresholds for ordinal data 4

A3 Structural equations of the models 6

A4 Measurement equations 12

A4.1 M1 . . . 12

A4.2 M7 . . . 14

A5 Summary of the constraints applied to parameters 16

A6 Software 16

A7 Results omitted in the main text 17

A8 Info about data collection 22

A9 Comparison with previous studies 24

A9.1 Comparison with Komdeur 1992 . . . 26

A9.2 Comparison with Brouwer et al. 2009 . . . 31

Bibliography 35

LIST OF FIGURES

A1 Graphical representation of a hypothetical ordinal categorical variable . . . 5

A2 Graphical representation of the indirect effect of TQ on R . . . 15

A3 Forestplots . . . 21

A4 Years of data analyzed by previous studies . . . 26

A5 Results of Komdeur 1992 . . . 28

A6 Results of Brouwer et al. 2009 . . . 32

LIST OF TABLES

A1 List of variables included in the models . . . 4

A2 Summary of the constraints applied to parameters in the SEMs 16
A3 Threshold estimates for the ordinal variables 17
A4 Model rankings with the $L_{\nu=0.5}-$ and $L_{\nu=0.8}-$measure 18
A5 GLM models ranking replicating Komdeur 1992 29
A6 Intercepts and contrasts for GLM replicating Komdeur 1992 30
A7 GLMMs rankings replicating Brouwer et al. 2009 33
A8 Intercepts and slopes for GLMMs replicating Brouwer et al. 2009 34

A1 ADDITIONAL INFORMATION ABOUT THE VARIABLES CONSIDERED

Variable name	LV	ID	Type	Levels	Categories
number of offspring intra-group	R	1	ord	3	$0, 1, 2+$
number of offspring extra-group	R	2	ord	3	$0, 1, 2+$
number of offspring only one known parent	R	3	ord	3	$0, 1, 2+$
group size	SE	4	ord	3	$0, 1, 2+$
number of helpers	SE	5	ord	3	$0, 1, 2+$
territory size	TQ	6	ord	4	$\leq 1500,\ (1500, 2500],$ $(2500, 3500],\ > 3500\, m^2$
insectXvegetation	TQ	7	ord	5	$\leq 3,\ (3, 6],$ $(6, 11],(11, 20],$ $> 20\ \frac{\#insect}{dm^2}$
age, age^2	fc		cont	-	

Table A1: List of variables included in the models. For each observed variable, we report the corresponding latent variable (LV) or if the observed variable is treated as a fixed covariate (fc). R stands for reproductive potential, SE for social environment and TQ for territory quality. The ID is a sequential number ID useful to interpret the JAGS scripts. The type denotes if a variable is ordinal (ord) or continuous (cont). When applicable, we report also the number of categories (levels) and the thresholds between categories. Open intervals are denoted with parentheses, while closed intervals are denoted with square brackets.

A2 CALCULATION OF THRESHOLDS FOR ORDINAL DATA

The ordinal variable $z_{♀,♂ io}$ was transformed into its continuous underlying variable $z'_{♀,♂ io} \sim N[0, \sigma^2]$ (Gelman et al. 2014; Lee 2007; Thanoon and Adnan 2015). To do so, we specified appropriate threshold values that relate $z_{♀,♂ io}$ to $z'_{♀,♂ io}$ (Fig. A1). For example, given an ordinal variable z including a categories, then

$$z = a \quad \text{if} \quad \alpha_{a-1} < x' \leq \alpha_a \tag{1}$$

where $z \in \{1, 2, \cdots, a\}$. The vector $\boldsymbol{\alpha} = \{\alpha_0, \alpha_1, \cdots, \alpha_a\}$ includes the thresholds and $\alpha_0 = -\infty$, $\alpha_a = \infty$. To avoid identification problems, the values of $\boldsymbol{\alpha}$ are set to constants and estimated through MCMC samples. To make sure the underlying continuous variables in the females and males have the same scale and are comparable we set identical values of $\boldsymbol{\alpha}$ in both samples (Song et al. 2011).

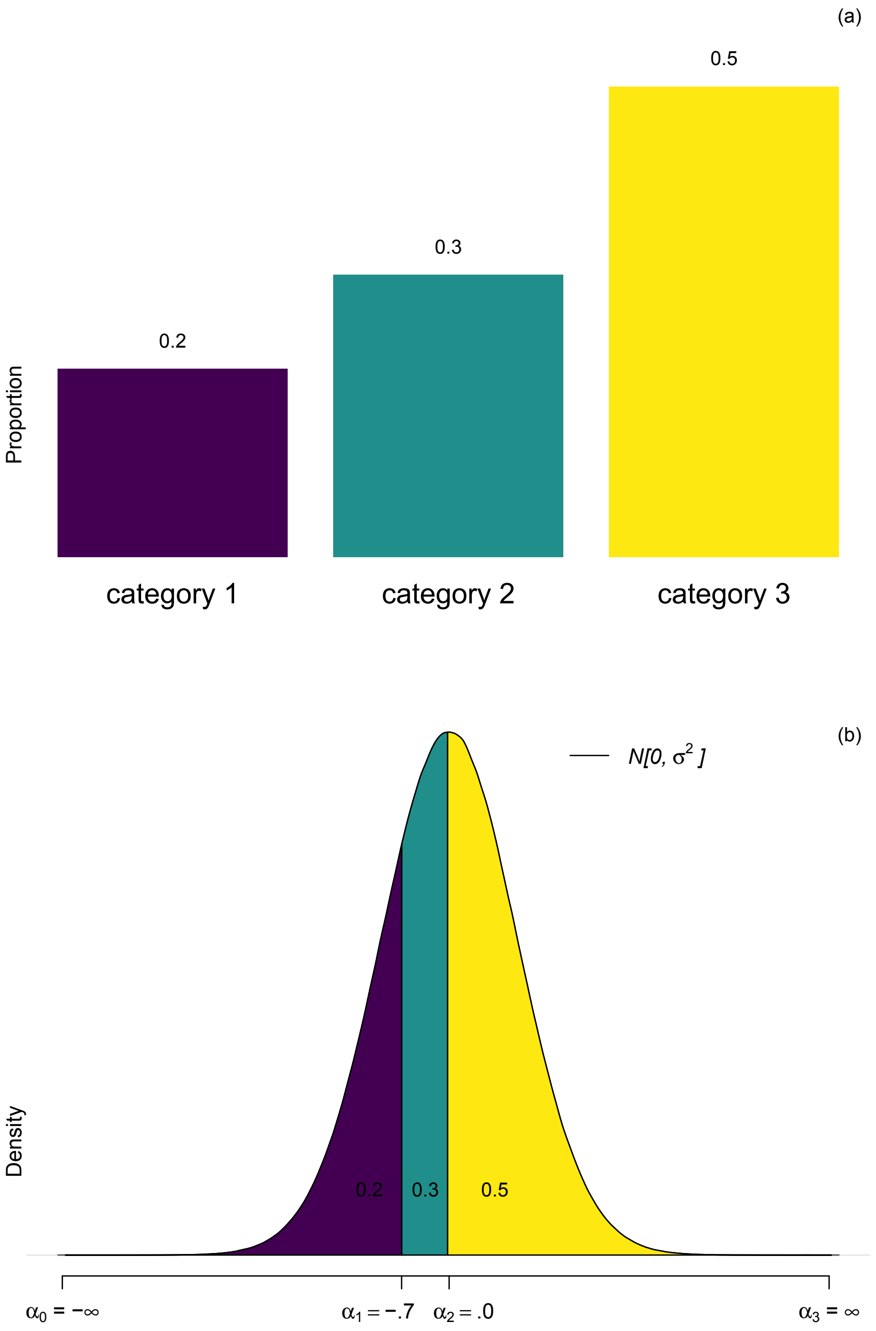

Figure A1: Graphical representation of a hypothetical ordinal categorical variable z with three categories. Panel (a) represents the proportions of the three categories in the variable. Panel (b) represents the underlying continuous variable, $z' \sim N[0, 1]$. The values of $\alpha_0, \cdots, \alpha_3$ are the appropriate thresholds that relate z to z'. From a practical perspective, the sampler substitutes each data point within the categorical variable with a random value between the corresponding thresholds. For example, a data point equal to category two is substituted with a random value within -0.7 and zero $(-0.7, 0]$.

A3 STRUCTURAL EQUATIONS OF THE MODELS

The two-sample three-level structural model for the reproductive success of females and males is given for each observation o of individual i nested in a social group s by

$$\begin{aligned} \boldsymbol{y}_{♀io} =& \boldsymbol{\Lambda}_{♀1io}(\boldsymbol{b}_{♀1io}\boldsymbol{\zeta}_{♀1io} + (\boldsymbol{I} - \boldsymbol{\Pi}_{♀1})^{-1}\boldsymbol{\Gamma}_{♀1}\boldsymbol{F}_1(\boldsymbol{\xi}_{♀1io}) + \boldsymbol{\delta}_{♀1io}) + \boldsymbol{\Lambda}_{♀1i\xi}\boldsymbol{\xi}_{♀1io} + \boldsymbol{\epsilon}_{♀1io} + \\ & \boldsymbol{\mu}_{♀} + \boldsymbol{\Lambda}_{♀2\eta}((\boldsymbol{I} - \boldsymbol{\Pi}_{♀2})^{-1}\boldsymbol{\Gamma}_{♀2}\boldsymbol{F}_2(\boldsymbol{\xi}_{♀2i}) + \boldsymbol{\delta}_{♀2i}) + \boldsymbol{\Lambda}_{♀2\xi}\boldsymbol{\xi}_{♀2i} + \boldsymbol{\epsilon}_{♀2i} + \\ & \boldsymbol{\mu}_s + \boldsymbol{\Lambda}_{s\eta}(\boldsymbol{\Gamma}_s\boldsymbol{F}_2(\boldsymbol{\xi}_s) + \boldsymbol{\delta}_s) + \boldsymbol{\Lambda}_{s\xi}\boldsymbol{F}_2(\boldsymbol{\xi}_s) + \boldsymbol{\epsilon}_s \end{aligned} \tag{2}$$

and

$$\begin{aligned} \boldsymbol{y}_{♂io} =& \boldsymbol{\Lambda}_{♂1io}(\boldsymbol{b}_{♂1io}\boldsymbol{\zeta}_{♂1io} + (\boldsymbol{I} - \boldsymbol{\Pi}_{♂1})^{-1}\boldsymbol{\Gamma}_{♂1}\boldsymbol{F}_1(\boldsymbol{\xi}_{♂1io}) + \boldsymbol{\delta}_{♂1io}) + \boldsymbol{\Lambda}_{♂1i\xi}\boldsymbol{\xi}_{♂1io} + \boldsymbol{\epsilon}_{♂1io} + \\ & \boldsymbol{\mu}_{♂} + \boldsymbol{\Lambda}_{♂2\eta}((\boldsymbol{I} - \boldsymbol{\Pi}_{♂2})^{-1}\boldsymbol{\Gamma}_{♂2}\boldsymbol{F}_2(\boldsymbol{\xi}_{♂2i}) + \boldsymbol{\delta}_{♂2i}) + \boldsymbol{\Lambda}_{♂2\xi}\boldsymbol{\xi}_{♂2i} + \boldsymbol{\epsilon}_{♂2i} + \\ & \boldsymbol{\mu}_s + \boldsymbol{\Lambda}_{s\eta}(\boldsymbol{\Gamma}_s\boldsymbol{F}_2(\boldsymbol{\xi}_s) + \boldsymbol{\delta}_s) + \boldsymbol{\Lambda}_{s\xi}\boldsymbol{F}_2(\boldsymbol{\xi}_s) + \boldsymbol{\epsilon}_s \end{aligned} \tag{3}$$

where, with regard to the female (♀) sample, $\boldsymbol{y}_{♀io}$ is the vector of observed variables; $\boldsymbol{\Lambda}_{♀2}$, $\boldsymbol{\Lambda}_{♀1i}$ and $\boldsymbol{\Lambda}_s$ are the matrix of factor loadings at the within-individual, between-individual, and social group level respectively and can be defined as $\boldsymbol{\Lambda}_{♀2} = (\boldsymbol{\Lambda}_{♀2\eta}, \boldsymbol{\Lambda}_{♀2\xi})$, $\boldsymbol{\Lambda}_{♀1i} = (\boldsymbol{\Lambda}_{♀1i}\eta, \boldsymbol{\Lambda}_{♀1i}\xi)$ and $\boldsymbol{\Lambda}_s = (\boldsymbol{\Lambda}_{s\eta}, \boldsymbol{\Lambda}_{s\xi})$ where the subscript η refers to the endogenous latent variables, and the subscript ξ refers to the exogenous latent variables; $\boldsymbol{b}_{♀}$ is the vector of regression coefficients; $\zeta_{♀}$ is the matrix of fixed covariates; $\boldsymbol{\Gamma}_{♀2}$, $\boldsymbol{\Gamma}_{♀1}$, $\boldsymbol{\Pi}_{♀1}$, and $\boldsymbol{\Pi}_{♀2}$ are matrices of unknown parameters; the values of $\boldsymbol{\Gamma}_s$ were set to be independent of $\boldsymbol{F}_2(\boldsymbol{\xi}_s)$ because the social-group level was merely introduced to correct for pseudoreplication and we wanted to avoid overfitting the model; $\boldsymbol{\xi}_{♀2i}$, $\boldsymbol{\xi}_{♀1io}$ and $\boldsymbol{\xi}_s$ are matrices of exogenous latent variables; $\boldsymbol{F}_2(\boldsymbol{\xi}_{♀2i}) =$ and $\boldsymbol{F}_1(\boldsymbol{\xi}_{♀1io})$ are vector-values functions transforming the values of $\boldsymbol{\xi}_{♀2i}$ and $\boldsymbol{\xi}_{♀1io}$ respectively; $\boldsymbol{\mu}_{♀}$ and $\boldsymbol{\mu}_s$ are vectors of intercepts; $\boldsymbol{\delta}_{♀2i} \sim N[\mathbf{0}, \boldsymbol{\Psi}_{♀2\delta}]$, $\boldsymbol{\delta}_{♀1i\delta} \sim N[\mathbf{0}, \boldsymbol{\Psi}_{♀1io\delta}]$ and $\boldsymbol{\delta}_s \sim N[\mathbf{0}, \boldsymbol{\Psi}_s]$ are vectors of measurement errors where $\boldsymbol{\Psi}_{♀2\delta}$, $\boldsymbol{\Psi}_{♀1i\delta}$ and $\boldsymbol{\Psi}_s$ are diagonal matrices; $\boldsymbol{\epsilon}_{♀2i} \sim N[\mathbf{0}, \boldsymbol{\Psi}_{♀2}]$, $\boldsymbol{\epsilon}_{♀1io} \sim N[\mathbf{0}, \boldsymbol{\Psi}_{♀1i}]$ and $\boldsymbol{\epsilon}_s \sim N[\mathbf{0}, \boldsymbol{\Psi}_s]$ are vectors of measurement errors where $\boldsymbol{\Psi}_{♀2}$, $\boldsymbol{\Psi}_{♀1i}$ and $\boldsymbol{\Psi}_s$ are diagonal matrix; and $\boldsymbol{I}$ is the identity matrix.

The measurement errors within-, between-individual and between-groups are assumed to be independent. All the vectors (bold-face lowercase letters) and matrices (bold-face uppercase letters) for the male sample are indicated by the same letters of the female sample but include the ♂ subscript. To solve the variance-covariance matrix of the model above defined it is necessary to impose some constraints on the parameters (Lee and Song 2012; Song et al.

2011). For example, some values of $\mathbf{\Lambda}_{\female,\male 1i}$ and $\mathbf{\Lambda}_{\female,\male 2}$ are set to 1 (see Fig. 1). This practice does not impact the interpretation of the model but allows to include variables with a different unit of measurement as an expression of the same latent variable (Cubaynes et al. 2012).

Equations for each model consired are reported below:

- $M1, M2, M3:$

$$\begin{bmatrix}\eta_{\female 1io,1}\end{bmatrix} = \begin{bmatrix}b_{1i,1} & b_{1i,2}\end{bmatrix} \times \begin{bmatrix}\zeta_{\female 1io,1} \\ \zeta_{\female 1io,2}\end{bmatrix} + \begin{bmatrix}\gamma_{\female 1i,11} & \gamma_{\female 1i,12}\end{bmatrix} \times \begin{bmatrix}\xi_{\female 1io,1} \\ \xi_{\female 1io,2}\end{bmatrix} + \begin{bmatrix}\delta_{\female 1io,1}\end{bmatrix}$$

$$\begin{bmatrix}\eta_{\female 2i,1}\end{bmatrix} = \begin{bmatrix}\gamma_{\female 2,11} & \gamma_{\female 2,12}\end{bmatrix} \times \begin{bmatrix}\xi_{\female 2i,1} \\ \xi_{\female 2i,2}\end{bmatrix} + \begin{bmatrix}\delta_{\female 2i,1}\end{bmatrix}$$

$$\begin{bmatrix}\eta_{\male 1io,1}\end{bmatrix} = \begin{bmatrix}b_{1i,1} & b_{1i,2}\end{bmatrix} \times \begin{bmatrix}\zeta_{\male 1io,1} \\ \zeta_{\male 1io,2}\end{bmatrix} + \begin{bmatrix}\gamma_{\male 1i,11} & \gamma_{\male 1i,12}\end{bmatrix} \times \begin{bmatrix}\xi_{\male 1io,1} \\ \xi_{\male 1io,2}\end{bmatrix} + \begin{bmatrix}\delta_{\male 1io,1}\end{bmatrix}$$

$$\begin{bmatrix}\eta_{\male 2i,1}\end{bmatrix} = \begin{bmatrix}\gamma_{\male 2,11} & \gamma_{\male 2,12}\end{bmatrix} \times \begin{bmatrix}\xi_{\male 2i,1} \\ \xi_{\male 2i,2}\end{bmatrix} + \begin{bmatrix}\delta_{\male 2i,1}\end{bmatrix}$$

$$\begin{bmatrix}\eta_{s,1}\end{bmatrix} = \begin{bmatrix}\delta_{s,1}\end{bmatrix}$$

- $M4:$

$$\begin{bmatrix}\eta_{\female 1io,1}\end{bmatrix} = \begin{bmatrix}b_{1i,1} & b_{1i,2}\end{bmatrix} \times \begin{bmatrix}\zeta_{\female 1io,1} \\ \zeta_{\female 1io,2}\end{bmatrix} + \begin{bmatrix}\gamma_{1i,11} & \gamma_{\female 1i,12}\end{bmatrix} \times \begin{bmatrix}\xi_{\female 1io,1} \\ \xi_{\female 1io,2}\end{bmatrix} + \begin{bmatrix}\delta_{\female 1io,1}\end{bmatrix}$$

$$\begin{bmatrix}\eta_{\female 2i,1}\end{bmatrix} = \begin{bmatrix}\gamma_{2,11} & \gamma_{\female 2,12}\end{bmatrix} \times \begin{bmatrix}\xi_{\female 2i,1} \\ \xi_{\female 2i,2}\end{bmatrix} + \begin{bmatrix}\delta_{\female 2i,1}\end{bmatrix}$$

$$\begin{bmatrix}\eta_{\male 1io,1}\end{bmatrix} = \begin{bmatrix}b_{1i,1} & b_{1i,2}\end{bmatrix} \times \begin{bmatrix}\zeta_{\male 1io,1} \\ \zeta_{\male 1io,2}\end{bmatrix} + \begin{bmatrix}\gamma_{1i,11} & \gamma_{\male 1i,12}\end{bmatrix} \times \begin{bmatrix}\xi_{\male 1io,1} \\ \xi_{\male 1io,2}\end{bmatrix} + \begin{bmatrix}\delta_{\male 1io,1}\end{bmatrix}$$

$$\begin{bmatrix}\eta_{\male 2i,1}\end{bmatrix} = \begin{bmatrix}\gamma_{2,11} & \gamma_{\male 2,12}\end{bmatrix} \times \begin{bmatrix}\xi_{\male 2i,1} \\ \xi_{\male 2i,2}\end{bmatrix} + \begin{bmatrix}\delta_{\male 2i,1}\end{bmatrix}$$

$$\begin{bmatrix}\eta_{s,1}\end{bmatrix} = \begin{bmatrix}\delta_{s,1}\end{bmatrix}$$

- $M5:$

$$\begin{bmatrix}\eta_{♀1io,1}\end{bmatrix} = \begin{bmatrix}b_{1i,1} & b_{1i,2}\end{bmatrix} \times \begin{bmatrix}\zeta_{♀1io,1}\\ \zeta_{♀1io,2}\end{bmatrix} + \begin{bmatrix}\gamma_{♀1i,11} & \gamma_{1i,12}\end{bmatrix} \times \begin{bmatrix}\xi_{♀1io,1}\\ \xi_{♀1io,2}\end{bmatrix} + \begin{bmatrix}\delta_{♀1io,1}\end{bmatrix}$$

$$\begin{bmatrix}\eta_{♀2i,1}\end{bmatrix} = \begin{bmatrix}\gamma_{♀2,11} & \gamma_{2,12}\end{bmatrix} \times \begin{bmatrix}\xi_{♀2i,1}\\ \xi_{♀2i,2}\end{bmatrix} + \begin{bmatrix}\delta_{♀2i,1}\end{bmatrix}$$

$$\begin{bmatrix}\eta_{♂1io,1}\end{bmatrix} = \begin{bmatrix}b_{1i,1} & b_{1i,2}\end{bmatrix} \times \begin{bmatrix}\zeta_{♂1io,1}\\ \zeta_{♂1io,2}\end{bmatrix} + \begin{bmatrix}\gamma_{♂1i,11} & \gamma_{1i,12}\end{bmatrix} \times \begin{bmatrix}\xi_{♂1io,1}\\ \xi_{♂1io,2}\end{bmatrix} + \begin{bmatrix}\delta_{♂1io,1}\end{bmatrix}$$

$$\begin{bmatrix}\eta_{♂2i,1}\end{bmatrix} = \begin{bmatrix}\gamma_{♂2,11} & \gamma_{2,12}\end{bmatrix} \times \begin{bmatrix}\xi_{♂2i,1}\\ \xi_{♂2i,2}\end{bmatrix} + \begin{bmatrix}\delta_{♂2i,1}\end{bmatrix}$$

$$\begin{bmatrix}\eta_{s,1}\end{bmatrix} = \begin{bmatrix}\delta_{s,1}\end{bmatrix}$$

- $M6:$

$$\begin{bmatrix}\eta_{♀1io,1}\end{bmatrix} = \begin{bmatrix}b_{1i,1} & b_{1i,2}\end{bmatrix} \times \begin{bmatrix}\zeta_{♀1io,1}\\ \zeta_{♀1io,2}\end{bmatrix} + \begin{bmatrix}\gamma_{1i,11} & \gamma_{1i,12}\end{bmatrix} \times \begin{bmatrix}\xi_{♀1io,1}\\ \xi_{♀1io,2}\end{bmatrix} + \begin{bmatrix}\delta_{♀1io,1}\end{bmatrix}$$

$$\begin{bmatrix}\eta_{♀2i,1}\end{bmatrix} = \begin{bmatrix}\gamma_{2,11} & \gamma_{2,12}\end{bmatrix} \times \begin{bmatrix}\xi_{♀2i,1}\\ \xi_{♀2i,2}\end{bmatrix} + \begin{bmatrix}\delta_{♀2i,1}\end{bmatrix}$$

$$\begin{bmatrix}\eta_{♂1io,1}\end{bmatrix} = \begin{bmatrix}b_{1i,1} & b_{1i,2}\end{bmatrix} \times \begin{bmatrix}\zeta_{♂1io,1}\\ \zeta_{♂1io,2}\end{bmatrix} + \begin{bmatrix}\gamma_{1i,11} & \gamma_{1i,12}\end{bmatrix} \times \begin{bmatrix}\xi_{♂1io,1}\\ \xi_{♂1io,2}\end{bmatrix} + \begin{bmatrix}\delta_{♂1io,1}\end{bmatrix}$$

$$\begin{bmatrix}\eta_{♂2i,1}\end{bmatrix} = \begin{bmatrix}\gamma_{2,11} & \gamma_{2,12}\end{bmatrix} \times \begin{bmatrix}\xi_{♂2i,1}\\ \xi_{♂2i,2}\end{bmatrix} + \begin{bmatrix}\delta_{♂2i,1}\end{bmatrix}$$

$$\begin{bmatrix}\eta_{s,1}\end{bmatrix} = \begin{bmatrix}\delta_{s,1}\end{bmatrix}$$

- $M7:$ $$\begin{bmatrix}\eta_{♀1io,1}\\ \eta_{♀1io,2}\end{bmatrix}=\begin{bmatrix}b_{1i,1} & b_{1i,2}\\ 0 & 0\end{bmatrix}\times\begin{bmatrix}\zeta_{♀1io,1}\\ \zeta_{♀1io,2}\end{bmatrix}+\begin{bmatrix}0 & \pi_{♀1,12}\\ 0 & 0\end{bmatrix}\times\begin{bmatrix}\eta_{♀1io,1}\\ \eta_{♀1io,2}\end{bmatrix}+\begin{bmatrix}0\\ \gamma_{♀1i,2}\end{bmatrix}\times\begin{bmatrix}\xi_{♀1io,1}\end{bmatrix}+\begin{bmatrix}\delta_{♀1io,1}\\ \delta_{♀1io,2}\end{bmatrix}$$
$$\begin{bmatrix}\eta_{♀2i,1}\\ \eta_{♀2i,2}\end{bmatrix}=\begin{bmatrix}0 & \pi_{♀2,12}\\ 0 & 0\end{bmatrix}\times\begin{bmatrix}\eta_{♀2i,1}\\ \eta_{♀2i,2}\end{bmatrix}+\begin{bmatrix}0\\ \gamma_{♀2,2}\end{bmatrix}\times\begin{bmatrix}\xi_{♀2i,1}\end{bmatrix}+\begin{bmatrix}\delta_{♀2i,1}\\ \delta_{♀2i,2}\end{bmatrix}$$
$$\begin{bmatrix}\eta_{♂1io,1}\\ \eta_{♂1io,2}\end{bmatrix}=\begin{bmatrix}b_{1i,1} & b_{1i,2}\\ 0 & 0\end{bmatrix}\times\begin{bmatrix}\zeta_{♂1io,1}\\ \zeta_{♂1io,2}\end{bmatrix}+\begin{bmatrix}0 & \pi_{♂1,12}\\ 0 & 0\end{bmatrix}\times\begin{bmatrix}\eta_{♂1io,1}\\ \eta_{♂1io,2}\end{bmatrix}+\begin{bmatrix}0\\ \gamma_{♂1i,2}\end{bmatrix}\times\begin{bmatrix}\xi_{♂1io,1}\end{bmatrix}+\begin{bmatrix}\delta_{♂1io,1}\\ \delta_{♂1io,2}\end{bmatrix}$$
$$\begin{bmatrix}\eta_{♂2i,1}\end{bmatrix}=\begin{bmatrix}0 & \pi_{♂2,12}\\ 0 & 0\end{bmatrix}\times\begin{bmatrix}\eta_{♂2i,1}\\ \eta_{♂2i,2}\end{bmatrix}+\begin{bmatrix}0\\ \gamma_{♂2,2}\end{bmatrix}\times\begin{bmatrix}\xi_{♂2i,1}\end{bmatrix}+\begin{bmatrix}\delta_{♂2i,1}\\ \delta_{♂2i,2}\end{bmatrix}$$
$$\begin{bmatrix}\eta_{s,1}\end{bmatrix}=\begin{bmatrix}\delta_{s,1}\end{bmatrix}$$

- $M8:$ $$\begin{bmatrix}\eta_{♀1io,1}\\ \eta_{♀1io,2}\end{bmatrix}=\begin{bmatrix}b_{1i,1} & b_{1i,2}\\ 0 & 0\end{bmatrix}\times\begin{bmatrix}\zeta_{♀1io,1}\\ \zeta_{♀1io,2}\end{bmatrix}+\begin{bmatrix}0 & \pi_{♀1,12}\\ 0 & 0\end{bmatrix}\times\begin{bmatrix}\eta_{♀1io,1}\\ \eta_{♀1io,2}\end{bmatrix}+\begin{bmatrix}0\\ \gamma_{1i,2}\end{bmatrix}\times\begin{bmatrix}\xi_{♀1io,1}\end{bmatrix}+\begin{bmatrix}\delta_{♀1io,1}\\ \delta_{♀1io,2}\end{bmatrix}$$
$$\begin{bmatrix}\eta_{♀2i,1}\\ \eta_{♀2i,2}\end{bmatrix}=\begin{bmatrix}0 & \pi_{♀2,12}\\ 0 & 0\end{bmatrix}\times\begin{bmatrix}\eta_{♀2i,1}\\ \eta_{♀2i,2}\end{bmatrix}+\begin{bmatrix}0\\ \gamma_{2,2}\end{bmatrix}\times\begin{bmatrix}\xi_{♀2i,1}\end{bmatrix}+\begin{bmatrix}\delta_{♀2i,1}\\ \delta_{♀2i,2}\end{bmatrix}$$
$$\begin{bmatrix}\eta_{♂1io,1}\\ \eta_{♂1io,2}\end{bmatrix}=\begin{bmatrix}b_{1i,1} & b_{1i,2}\\ 0 & 0\end{bmatrix}\times\begin{bmatrix}\zeta_{♂1io,1}\\ \zeta_{♂1io,2}\end{bmatrix}+\begin{bmatrix}0 & \pi_{♂1,12}\\ 0 & 0\end{bmatrix}\times\begin{bmatrix}\eta_{♂1io,1}\\ \eta_{♂1io,2}\end{bmatrix}+\begin{bmatrix}0\\ \gamma_{1i,2}\end{bmatrix}\times\begin{bmatrix}\xi_{♂1io,1}\end{bmatrix}+\begin{bmatrix}\delta_{♂1io,1}\\ \delta_{♂1io,2}\end{bmatrix}$$
$$\begin{bmatrix}\eta_{♂2i,1}\end{bmatrix}=\begin{bmatrix}0 & \pi_{♂2,12}\\ 0 & 0\end{bmatrix}\times\begin{bmatrix}\eta_{♂2i,1}\\ \eta_{♂2i,2}\end{bmatrix}+\begin{bmatrix}0\\ \gamma_{2,2}\end{bmatrix}\times\begin{bmatrix}\xi_{♂2i,1}\end{bmatrix}+\begin{bmatrix}\delta_{♂2i,1}\\ \delta_{♂2i,2}\end{bmatrix}$$
$$\begin{bmatrix}\eta_{s,1}\end{bmatrix}=\begin{bmatrix}\delta_{s,1}\end{bmatrix}$$

- $M9:$ $$\begin{bmatrix}\eta_{♀1io,1}\\ \eta_{♀1io,2}\end{bmatrix}=\begin{bmatrix}b_{1i,1} & b_{1i,2}\\ 0 & 0\end{bmatrix}\times\begin{bmatrix}\zeta_{♀1io,1}\\ \zeta_{♀1io,2}\end{bmatrix}+\begin{bmatrix}0 & \pi_{1,12}\\ 0 & 0\end{bmatrix}\times\begin{bmatrix}\eta_{♀1io,1}\\ \eta_{♀1io,2}\end{bmatrix}+\begin{bmatrix}0\\ \gamma_{♀1i,2}\end{bmatrix}\times\begin{bmatrix}\xi_{♀1io,1}\end{bmatrix}+\begin{bmatrix}\delta_{♀1io,1}\\ \delta_{♀1io,2}\end{bmatrix}$$
$$\begin{bmatrix}\eta_{♀2i,1}\\ \eta_{♀2i,2}\end{bmatrix}=\begin{bmatrix}0 & \pi_{2,12}\\ 0 & 0\end{bmatrix}\times\begin{bmatrix}\eta_{♀2i,1}\\ \eta_{♀2i,2}\end{bmatrix}+\begin{bmatrix}0\\ \gamma_{♀2,2}\end{bmatrix}\times\begin{bmatrix}\xi_{♀2i,1}\end{bmatrix}+\begin{bmatrix}\delta_{♀2i,1}\\ \delta_{♀2i,2}\end{bmatrix}$$
$$\begin{bmatrix}\eta_{♂1io,1}\\ \eta_{♂1io,2}\end{bmatrix}=\begin{bmatrix}b_{1i,1} & b_{1i,2}\\ 0 & 0\end{bmatrix}\times\begin{bmatrix}\zeta_{♂1io,1}\\ \zeta_{♂1io,2}\end{bmatrix}+\begin{bmatrix}0 & \pi_{1,12}\\ 0 & 0\end{bmatrix}\times\begin{bmatrix}\eta_{♂1io,1}\\ \eta_{♂1io,2}\end{bmatrix}+\begin{bmatrix}0\\ \gamma_{♂1i,2}\end{bmatrix}\times\begin{bmatrix}\xi_{♂1io,1}\end{bmatrix}+\begin{bmatrix}\delta_{♂1io,1}\\ \delta_{♂1io,2}\end{bmatrix}$$
$$\begin{bmatrix}\eta_{♂2i,1}\end{bmatrix}=\begin{bmatrix}0 & \pi_{2,12}\\ 0 & 0\end{bmatrix}\times\begin{bmatrix}\eta_{♂2i,1}\\ \eta_{♂2i,2}\end{bmatrix}+\begin{bmatrix}0\\ \gamma_{♂2,2}\end{bmatrix}\times\begin{bmatrix}\xi_{♂2i,1}\end{bmatrix}+\begin{bmatrix}\delta_{♂2i,1}\\ \delta_{♂2i,2}\end{bmatrix}$$
$$\begin{bmatrix}\eta_{s,1}\end{bmatrix}=\begin{bmatrix}\delta_{s,1}\end{bmatrix}$$
- $M10:$ $$\begin{bmatrix}\eta_{♀1io,1}\\ \eta_{♀1io,2}\end{bmatrix}=\begin{bmatrix}b_{1i,1} & b_{1i,2}\\ 0 & 0\end{bmatrix}\times\begin{bmatrix}\zeta_{♀1io,1}\\ \zeta_{♀1io,2}\end{bmatrix}+\begin{bmatrix}0 & \pi_{1,12}\\ 0 & 0\end{bmatrix}\times\begin{bmatrix}\eta_{♀1io,1}\\ \eta_{♀1io,2}\end{bmatrix}+\begin{bmatrix}0\\ \gamma_{1i,2}\end{bmatrix}\times\begin{bmatrix}\xi_{♀1io,1}\end{bmatrix}+\begin{bmatrix}\delta_{♀1io,1}\\ \delta_{♀1io,2}\end{bmatrix}$$
$$\begin{bmatrix}\eta_{♀2i,1}\\ \eta_{♀2i,2}\end{bmatrix}=\begin{bmatrix}0 & \pi_{2,12}\\ 0 & 0\end{bmatrix}\times\begin{bmatrix}\eta_{♀2i,1}\\ \eta_{♀2i,2}\end{bmatrix}+\begin{bmatrix}0\\ \gamma_{2,2}\end{bmatrix}\times\begin{bmatrix}\xi_{♀2i,1}\end{bmatrix}+\begin{bmatrix}\delta_{♀2i,1}\\ \delta_{♀2i,2}\end{bmatrix}$$
$$\begin{bmatrix}\eta_{♂1io,1}\\ \eta_{♂1io,2}\end{bmatrix}=\begin{bmatrix}b_{1i,1} & b_{1i,2}\\ 0 & 0\end{bmatrix}\times\begin{bmatrix}\zeta_{♂1io,1}\\ \zeta_{♂1io,2}\end{bmatrix}+\begin{bmatrix}0 & \pi_{1,12}\\ 0 & 0\end{bmatrix}\times\begin{bmatrix}\eta_{♂1io,1}\\ \eta_{♂1io,2}\end{bmatrix}+\begin{bmatrix}0\\ \gamma_{1i,2}\end{bmatrix}\times\begin{bmatrix}\xi_{♂1io,1}\end{bmatrix}+\begin{bmatrix}\delta_{♂1io,1}\\ \delta_{♂1io,2}\end{bmatrix}$$
$$\begin{bmatrix}\eta_{♂2i,1}\end{bmatrix}=\begin{bmatrix}0 & \pi_{2,12}\\ 0 & 0\end{bmatrix}\times\begin{bmatrix}\eta_{♂2i,1}\\ \eta_{♂2i,2}\end{bmatrix}+\begin{bmatrix}0\\ \gamma_{2,2}\end{bmatrix}\times\begin{bmatrix}\xi_{♂2i,1}\end{bmatrix}+\begin{bmatrix}\delta_{♂2i,1}\\ \delta_{♂2i,2}\end{bmatrix}$$
$$\begin{bmatrix}\eta_{s,1}\end{bmatrix}=\begin{bmatrix}\delta_{s,1}\end{bmatrix}$$

where

- $\eta_{♀1io,1}, \eta_{♀2i,1}$ = female reproductive potential within- and between-individual,

- $\xi_{♀1io,1}, \xi_{♀2i,1}$ = female social environment within- and between-individual,
- $\xi_{♀1io,2}, \xi_{♀2i,2}$ = female territory quality within- and between-individual,
- $\eta_{s,1}$ = reproductive potential between social group.
- note that in $\boldsymbol{M7}$ to $\boldsymbol{M10}$: $\eta_{♀1io,2}, \eta_{♀2i,2}$ = indicate the female social environment within- and between-individual, while $\xi_{♀1io,1}, \xi_{♀2i,1}$ = indicate the female territory quality within- and between-individual,
- Similar definitions apply to the male sample (replace the ♀ symbol with the ♂ symbol).

A4 MEASUREMENT EQUATIONS

A4.1 M1

$$
\begin{bmatrix} y_{\female io,1} \\ y_{\female io,2} \\ y_{\female io,3} \\ y_{\female io,4} \\ y_{\female io,5} \\ y_{\female io,6} \\ y_{\female io,7} \end{bmatrix} = \begin{bmatrix} \mu_{\female 1} \\ \mu_{\female 2} \\ \mu_{\female 3} \\ \mu_{\female 4} \\ \mu_{\female 5} \\ \mu_{\female 6} \\ \mu_{\female 7} \end{bmatrix} + \begin{bmatrix} 1 & 0 & 0 \\ \lambda_{\female 2,2-1} & 0 & 0 \\ \lambda_{\female 2,3-1} & 0 & 0 \\ 0 & 1 & 0 \\ 0 & \lambda_{\female 2,5-2} & 0 \\ 0 & 0 & 1 \\ 0 & 0 & \lambda_{\female 2,7-3} \end{bmatrix} \times \begin{bmatrix} \eta_{\female 2i,1} \\ \xi_{\female 2i,1} \\ \xi_{\female 2i,2} \end{bmatrix} + \begin{bmatrix} \epsilon_{\female 2i,1} \\ \epsilon_{\female 2i,2} \\ \epsilon_{\female 2i,3} \\ \epsilon_{\female 2i,4} \\ \epsilon_{\female 2i,5} \\ \epsilon_{\female 2i,6} \\ \epsilon_{\female 2i,7} \\ \epsilon_{\female 2i,8} \\ \epsilon_{\female 2i,9} \end{bmatrix} + \begin{bmatrix} b_{1io,11} & b_{1io,12} \\ b_{1io,21} & b_{1io,22} \\ b_{1io,31} & b_{1io,32} \\ b_{1io,41} & b_{1io,42} \\ b_{1io,51} & b_{1io,52} \\ b_{1io,61} & b_{1io,62} \\ b_{\female 1io,71} & b_{1io,72} \end{bmatrix} \times \begin{bmatrix} \zeta_{\female 1io,1} \\ \zeta_{\female 1io,2} \end{bmatrix} + \begin{bmatrix} 1 & 0 & 0 \\ \lambda_{\female 1i,2-1} & 0 & 0 \\ \lambda_{\female 1i,3-1} & 0 & 0 \\ 0 & 1 & 0 \\ 0 & \lambda_{\female 1i,5-2} & 0 \\ 0 & 0 & 1 \\ 0 & 0 & \lambda_{\female 1i,7-3} \end{bmatrix} \times \begin{bmatrix} \eta_{\female 1io,1} \\ \xi_{\female 1io,1} \\ \xi_{\female 1io,2} \end{bmatrix} + \begin{bmatrix} \epsilon_{\female 1io,1} \\ \epsilon_{\female 1io,2} \\ \epsilon_{\female 1io,3} \\ \epsilon_{\female 1io,4} \\ \epsilon_{\female 1io,5} \\ \epsilon_{\female 1io,6} \\ \epsilon_{\female 1io,7} \\ \epsilon_{\female 1io,8} \\ \epsilon_{\female 1io,9} \end{bmatrix} + \begin{bmatrix} 1 & 0 & 0 \\ \lambda_{s,2-1} & 0 & 0 \\ \lambda_{s,3-1} & 0 & 0 \\ 0 & 1 & 0 \\ 0 & \lambda_{s,5-2} & 0 \\ 0 & 0 & 1 \\ 0 & 0 & \lambda_{s,7-3} \end{bmatrix} \times \begin{bmatrix} \eta_{s,1} \\ \xi_{s,1} \\ \xi_{s,2} \end{bmatrix} + \begin{bmatrix} \epsilon_{s,1} \\ \epsilon_{s,2} \\ \epsilon_{s,3} \\ \epsilon_{s,4} \\ \epsilon_{s,5} \\ \epsilon_{s,6} \\ \epsilon_{s,7} \end{bmatrix}
$$

$$
\begin{bmatrix} y_{\male io,1} \\ y_{\male io,2} \\ y_{\male io,3} \\ y_{\male io,4} \\ y_{\male io,5} \\ y_{\male io,6} \\ y_{\male io,7} \end{bmatrix} = \begin{bmatrix} \mu_{\male 1} \\ \mu_{\male 2} \\ \mu_{\male 3} \\ \mu_{\male 4} \\ \mu_{\male 5} \\ \mu_{\male 6} \\ \mu_{\male 7} \end{bmatrix} + \begin{bmatrix} 1 & 0 & 0 \\ \lambda_{\male 2,2-1} & 0 & 0 \\ \lambda_{\male 2,3-1} & 0 & 0 \\ 0 & 1 & 0 \\ 0 & \lambda_{\male 2,5-2} & 0 \\ 0 & 0 & 1 \\ 0 & 0 & \lambda_{\male 2,7-3} \end{bmatrix} \times \begin{bmatrix} \eta_{\male 2i,1} \\ \xi_{\male 2i,1} \\ \xi_{\male 2i,2} \end{bmatrix} + \begin{bmatrix} \epsilon_{\male 2i,1} \\ \epsilon_{\male 2i,2} \\ \epsilon_{\male 2i,3} \\ \epsilon_{\male 2i,4} \\ \epsilon_{\male 2i,5} \\ \epsilon_{\male 2i,6} \\ \epsilon_{\male 2i,7} \end{bmatrix} + \begin{bmatrix} b_{1io,11} & b_{1io,12} \\ b_{1io,21} & b_{1io,22} \\ b_{1io,31} & b_{1io,32} \\ b_{1io,41} & b_{1io,42} \\ b_{1io,51} & b_{1io,52} \\ b_{1io,61} & b_{1io,62} \\ b_{\male 1io,71} & b_{1io,72} \end{bmatrix} \times \begin{bmatrix} \zeta_{\male 1io,1} \\ \zeta_{\male 1io,2} \end{bmatrix} + \begin{bmatrix} 1 & 0 & 0 \\ \lambda_{\male 1i,2-1} & 0 & 0 \\ \lambda_{\male 1i,3-1} & 0 & 0 \\ 0 & 1 & 0 \\ 0 & \lambda_{\male 1i,5-2} & 0 \\ 0 & 0 & 1 \\ 0 & 0 & \lambda_{\male 1i,7-3} \end{bmatrix} \times \begin{bmatrix} \eta_{\male 1io,1} \\ \xi_{\male 1io,1} \\ \xi_{\male 1io,2} \end{bmatrix} + \begin{bmatrix} \epsilon_{\male 1io,1} \\ \epsilon_{\male 1io,2} \\ \epsilon_{\male 1io,3} \\ \epsilon_{\male 1io,4} \\ \epsilon_{\male 1io,5} \\ \epsilon_{\male 1io,6} \\ \epsilon_{\male 1io,7} \end{bmatrix} + \begin{bmatrix} 1 & 0 & 0 \\ \lambda_{s,2-1} & 0 & 0 \\ \lambda_{s,3-1} & 0 & 0 \\ 0 & 1 & 0 \\ 0 & \lambda_{s,5-2} & 0 \\ 0 & 0 & 1 \\ 0 & 0 & \lambda_{s,7-3} \end{bmatrix} \times \begin{bmatrix} \eta_{s,1} \\ \xi_{s,1} \\ \xi_{s,2} \end{bmatrix} + \begin{bmatrix} \epsilon_{s,1} \\ \epsilon_{s,2} \\ \epsilon_{s,3} \\ \epsilon_{s,4} \\ \epsilon_{s,5} \\ \epsilon_{s,6} \\ \epsilon_{s,7} \end{bmatrix} \tag{4}
$$

The measurement equations of the models $M2$ to $M6$ can be easily derived from those of $M1$ by setting the relevant parameters to be invariant (see Table A2).

A4.2 M7

$$
\begin{bmatrix} y_{\text{♀}io,1} \\ y_{\text{♀}io,2} \\ y_{\text{♀}io,3} \\ y_{\text{♀}io,4} \\ y_{\text{♀}io,5} \\ y_{\text{♀}io,6} \\ y_{\text{♀}io,7} \end{bmatrix}
=
\begin{bmatrix} \mu_{\text{♀}1} \\ \mu_{\text{♀}2} \\ \mu_{\text{♀}3} \\ \mu_{\text{♀}4} \\ \mu_{\text{♀}5} \\ \mu_{\text{♀}6} \\ \mu_{\text{♀}7} \end{bmatrix}
+
\begin{bmatrix} 1 & 0 & 0 \\ \lambda_{\text{♀}2,2-1} & 0 & 0 \\ \lambda_{\text{♀}2,3-1} & 0 & 0 \\ 0 & 1 & 0 \\ 0 & \lambda_{\text{♀}2,5-2} & 0 \\ 0 & 0 & 1 \\ 0 & 0 & \lambda_{\text{♀}2,7-3} \end{bmatrix}
\times
\begin{bmatrix} \eta_{\text{♀}2i,1} \\ \eta_{\text{♀}2i,2} \\ \xi_{\text{♀}2i,1} \end{bmatrix}
+
\begin{bmatrix} \epsilon_{\text{♀}2i,1} \\ \epsilon_{\text{♀}2i,2} \\ \epsilon_{\text{♀}2i,3} \\ \epsilon_{\text{♀}2i,4} \\ \epsilon_{\text{♀}2i,5} \\ \epsilon_{\text{♀}2i,6} \\ \epsilon_{\text{♀}2i,7} \\ \epsilon_{\text{♀}2i,8} \\ \epsilon_{\text{♀}2i,9} \end{bmatrix}
+
\begin{bmatrix} b_{1io,11} & b_{1io,12} \\ b_{1io,21} & b_{1io,22} \\ b_{1io,31} & b_{1io,32} \\ b_{1io,41} & b_{1io,42} \\ b_{1io,51} & b_{1io,52} \\ b_{1io,61} & b_{1io,62} \\ b_{1io,71} & b_{1io,72} \end{bmatrix}
\times
\begin{bmatrix} \zeta_{\text{♀}1io,1} \\ \zeta_{\text{♀}1io,2} \end{bmatrix}
+
\begin{bmatrix} 1 & 0 & 0 \\ \lambda_{\text{♀}1i,2-1} & 0 & 0 \\ \lambda_{\text{♀}1i,3-1} & 0 & 0 \\ 0 & 1 & 0 \\ 0 & \lambda_{\text{♀}1i,5-2} & 0 \\ 0 & 0 & 1 \\ 0 & 0 & \lambda_{\text{♀}1i,7-3} \end{bmatrix}
\times
\begin{bmatrix} \eta_{\text{♀}1io,1} \\ \eta_{\text{♀}1io,2} \\ \xi_{\text{♀}1io,1} \end{bmatrix}
+
\begin{bmatrix} \epsilon_{\text{♀}1io,1} \\ \epsilon_{\text{♀}1io,2} \\ \epsilon_{\text{♀}1io,3} \\ \epsilon_{\text{♀}1io,4} \\ \epsilon_{\text{♀}1io,5} \\ \epsilon_{\text{♀}1io,6} \\ \epsilon_{\text{♀}1io,7} \\ \epsilon_{\text{♀}1io,8} \\ \epsilon_{\text{♀}1io,9} \end{bmatrix}
+
\begin{bmatrix} 1 & 0 & 0 \\ \lambda_{s,2-1} & 0 & 0 \\ \lambda_{s,3-1} & 0 & 0 \\ 0 & 1 & 0 \\ 0 & \lambda_{s,5-2} & 0 \\ 0 & 0 & 1 \\ 0 & 0 & \lambda_{s,7-3} \end{bmatrix}
\times
\begin{bmatrix} \eta_{s,1} \\ \eta_{s,2} \\ \xi_{s,2} \end{bmatrix}
+
\begin{bmatrix} \epsilon_{s,1} \\ \epsilon_{s,2} \\ \epsilon_{s,3} \\ \epsilon_{s,4} \\ \epsilon_{s,5} \\ \epsilon_{s,6} \\ \epsilon_{s,7} \end{bmatrix}
$$

$$
\begin{bmatrix} y_{\text{♂}io,1} \\ y_{\text{♂}io,2} \\ y_{\text{♂}io,3} \\ y_{\text{♂}io,4} \\ y_{\text{♂}io,5} \\ y_{\text{♂}io,6} \\ y_{\text{♂}io,7} \end{bmatrix}
=
\begin{bmatrix} \mu_{\text{♂}1} \\ \mu_{\text{♂}2} \\ \mu_{\text{♂}3} \\ \mu_{\text{♂}4} \\ \mu_{\text{♂}5} \\ \mu_{\text{♂}6} \\ \mu_{\text{♂}7} \end{bmatrix}
+
\begin{bmatrix} 1 & 0 & 0 \\ \lambda_{\text{♂}2,2-1} & 0 & 0 \\ \lambda_{\text{♂}2,3-1} & 0 & 0 \\ 0 & 1 & 0 \\ 0 & \lambda_{\text{♂}2,5-2} & 0 \\ 0 & 0 & 1 \\ 0 & 0 & \lambda_{\text{♂}2,7-3} \end{bmatrix}
\times
\begin{bmatrix} \eta_{\text{♂}2i,1} \\ \eta_{\text{♂}2i,2} \\ \xi_{\text{♂}2i,1} \end{bmatrix}
+
\begin{bmatrix} \epsilon_{\text{♂}2i,1} \\ \epsilon_{\text{♂}2i,2} \\ \epsilon_{\text{♂}2i,3} \\ \epsilon_{\text{♂}2i,4} \\ \epsilon_{\text{♂}2i,5} \\ \epsilon_{\text{♂}2i,6} \\ \epsilon_{\text{♂}2i,7} \end{bmatrix}
+
\begin{bmatrix} b_{1io,11} & b_{1io,12} \\ b_{1io,21} & b_{1io,22} \\ b_{1io,31} & b_{1io,32} \\ b_{1io,41} & b_{1io,42} \\ b_{1io,51} & b_{1io,52} \\ b_{1io,61} & b_{1io,62} \\ b_{1io,71} & b_{1io,72} \end{bmatrix}
\times
\begin{bmatrix} \zeta_{\text{♂}1io,1} \\ \zeta_{\text{♂}1io,2} \end{bmatrix}
+
\begin{bmatrix} 1 & 0 & 0 \\ \lambda_{\text{♂}1i,2-1} & 0 & 0 \\ \lambda_{\text{♂}1i,3-1} & 0 & 0 \\ 0 & 1 & 0 \\ 0 & \lambda_{\text{♂}1i,5-2} & 0 \\ 0 & 0 & 1 \\ 0 & 0 & \lambda_{\text{♂}1i,7-3} \end{bmatrix}
\times
\begin{bmatrix} \eta_{\text{♂}1io,1} \\ \eta_{\text{♂}1io,2} \\ \xi_{\text{♂}1io,1} \end{bmatrix}
+
\begin{bmatrix} \epsilon_{\text{♂}1io,1} \\ \epsilon_{\text{♂}1io,2} \\ \epsilon_{\text{♂}1io,3} \\ \epsilon_{\text{♂}1io,4} \\ \epsilon_{\text{♂}1io,5} \\ \epsilon_{\text{♂}1io,6} \\ \epsilon_{\text{♂}1io,7} \end{bmatrix}
+
\begin{bmatrix} 1 & 0 & 0 \\ \lambda_{s,2-1} & 0 & 0 \\ \lambda_{s,3-1} & 0 & 0 \\ 0 & 1 & 0 \\ 0 & \lambda_{s,5-2} & 0 \\ 0 & 0 & 1 \\ 0 & 0 & \lambda_{s,7-3} \end{bmatrix}
\times
\begin{bmatrix} \eta_{s,1} \\ \eta_{s,2} \\ \xi_{s,1} \end{bmatrix}
+
\begin{bmatrix} \epsilon_{s,1} \\ \epsilon_{s,2} \\ \epsilon_{s,3} \\ \epsilon_{s,4} \\ \epsilon_{s,5} \\ \epsilon_{s,6} \\ \epsilon_{s,7} \end{bmatrix}
\quad (5)
$$

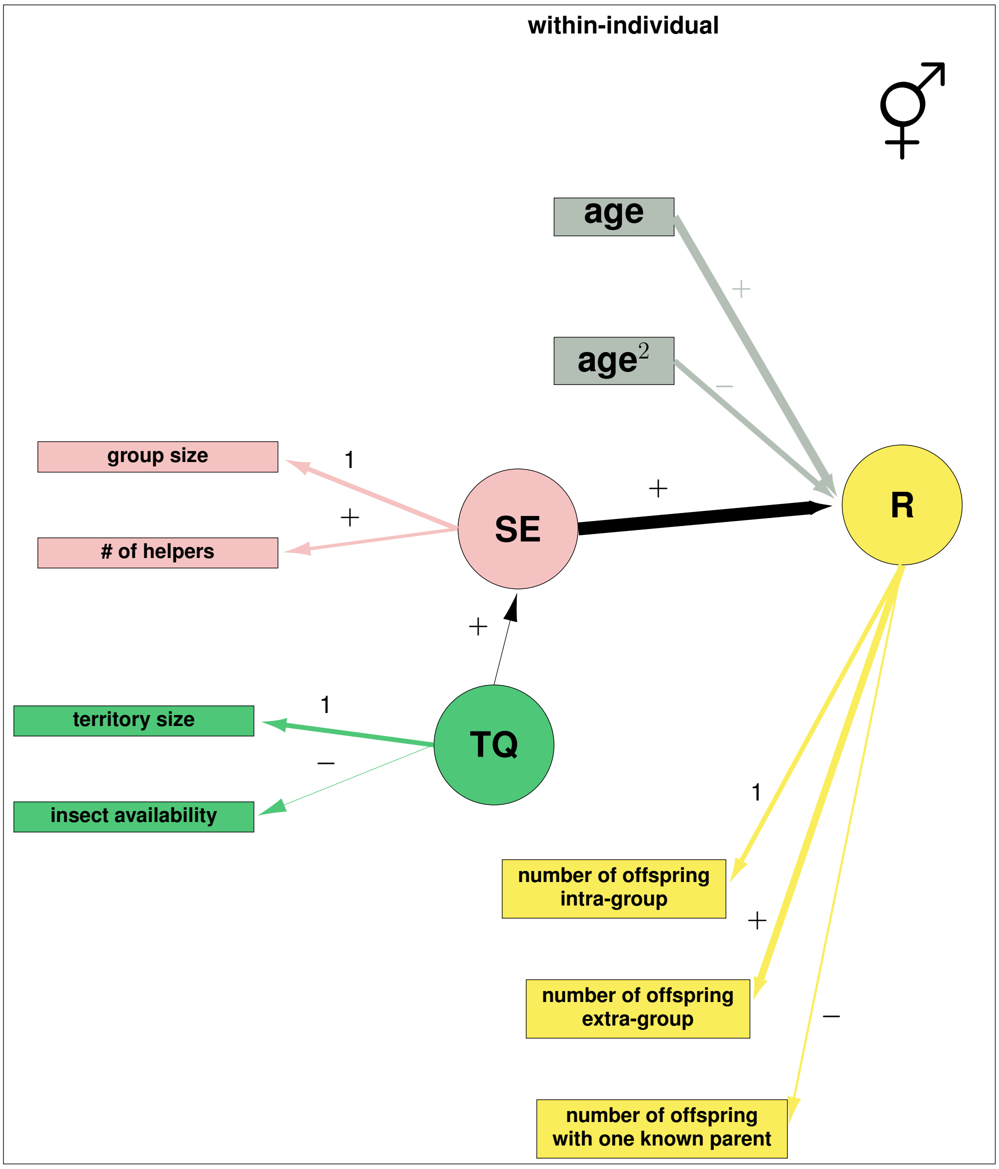

Figure A2: Graphical representation of the alternative path diagram in $M7, M8, M9$, and $M10$. Similar to Fig. 1, circles represent the latent variables (R = reproduction potential, SE = social environment, and TQ = territory quality), while rectangles represent fixed covariates and observed variables. To highlight the relationship between observed and latent variables, we colored variables related to the social environment in pink, to territory quality in green, and to reproduction in yellow. Fixed covariates (age, age^2) are colored in grey. The path represents both the female and male samples. This path describes an indirect effect of TQ on R. In fact, TQ is directly causing variations in the SE, which is then affecting R (TQ $\rightarrow$SE $\rightarrow$R). These relationships are present in models $M7$ to $M10$, but some of these lines are kept invariant between females and males in different models. For example, the relationships between TQ $\rightarrow$SE and SE $\rightarrow$R differ between females and males in models $M7$, while are invariant in models $M10$. The thickness of the lines is proportional to the relative importance of the corresponding parameter estimate in $M10$, where all the lines are invariant between females and males. The number 1 above the lines indicates that the corresponding observed variable was set as the reference level for the latent variable. The plus and minus signs at the top of each line indicate if the parameter is positive or negative.

A5 SUMMARY OF THE CONSTRAINTS APPLIED TO PARAMETERS

Model	Contraints on Parameters
M1	$\zeta_{♀} = \zeta_{♂}$
M2	$\mathbf{\Lambda}_{♀1i} = \mathbf{\Lambda}_{♂1i}$, $\mathbf{\Lambda}_{♀2} = \mathbf{\Lambda}_{♂2}$, and $\zeta_{♀} = \zeta_{♂}$
M3	$\mathbf{\Lambda}_{♀2} = \mathbf{\Lambda}_{♂2}$, $\mathbf{\Lambda}_{♀1i} = \mathbf{\Lambda}_{♂1i}$, $\boldsymbol{\mu}_{♀} = \boldsymbol{\mu}_{♂}$, and $\zeta_{♀} = \zeta_{♂}$
M4	$\mathbf{\Lambda}_{♀2} = \mathbf{\Lambda}_{♂2}$, $\mathbf{\Lambda}_{♀1i} = \mathbf{\Lambda}_{♂1i}$, $\boldsymbol{\mu}_{♀} = \boldsymbol{\mu}_{♂}$, $\mathbf{\Gamma}_{♀1} = \mathbf{\Gamma}_{♂1}$, $\mathbf{\Gamma}_{♀2} = \mathbf{\Gamma}_{♂2}$, and $\zeta_{♀} = \zeta_{♂}$
M5	$\mathbf{\Lambda}_{♀2} = \mathbf{\Lambda}_{♂2}$, $\mathbf{\Lambda}_{♀1i} = \mathbf{\Lambda}_{♂1i}$, $\boldsymbol{\mu}_{♀} = \boldsymbol{\mu}_{♂}$, $\mathbf{\Gamma}_{♀1} = \mathbf{\Gamma}_{♂1}$, $\mathbf{\Gamma}_{♀2} = \mathbf{\Gamma}_{♂2}$, and $\zeta_{♀} = \zeta_{♂}$
M6	$\mathbf{\Lambda}_{♀1i} = \mathbf{\Lambda}_{♂1i}$, $\mathbf{\Lambda}_{♀2} = \mathbf{\Lambda}_{♂2}$, $\boldsymbol{\mu}_{♀} = \boldsymbol{\mu}_{♂}$, $\mathbf{\Gamma}_{♀1} = \mathbf{\Gamma}_{♂1}$, $\mathbf{\Gamma}_{♀2} = \mathbf{\Gamma}_{♂2}$, and $\zeta_{♀} = \zeta_{♂}$
M7	$\mathbf{\Lambda}_{♀2} = \mathbf{\Lambda}_{♂2}$, $\mathbf{\Lambda}_{♀1i} = \mathbf{\Lambda}_{♂1i}$, $\boldsymbol{\mu}_{♀} = \boldsymbol{\mu}_{♂}$, and $\zeta_{♀} = \zeta_{♂}$
M8	$\mathbf{\Lambda}_{♀2} = \mathbf{\Lambda}_{♂2}$, $\mathbf{\Lambda}_{♀1i} = \mathbf{\Lambda}_{♂1i}$, $\boldsymbol{\mu}_{♀} = \boldsymbol{\mu}_{♂}$, $\mathbf{\Gamma}_{♀1} = \mathbf{\Gamma}_{♂1}$, $\mathbf{\Gamma}_{♀2} = \mathbf{\Gamma}_{♂2}$, and $\zeta_{♀} = \zeta_{♂}$
M9	$\mathbf{\Lambda}_{♀2} = \mathbf{\Lambda}_{♂2}$, $\mathbf{\Lambda}_{♀1i} = \mathbf{\Lambda}_{♂1i}$, $\boldsymbol{\mu}_{♀} = \boldsymbol{\mu}_{♂}$, $\mathbf{\Pi}_{♀1} = \mathbf{\Pi}_{♂1}$, $\mathbf{\Pi}_{♀2} = \mathbf{\Pi}_{♂2}$, and $\zeta_{♀} = \zeta_{♂}$
M10	$\mathbf{\Lambda}_{♀1i} = \mathbf{\Lambda}_{♂1i}$, $\mathbf{\Lambda}_{♀2} = \mathbf{\Lambda}_{♂2}$, $\boldsymbol{\mu}_{♀} = \boldsymbol{\mu}_{♂}$, $\mathbf{\Gamma}_{♀1} = \mathbf{\Gamma}_{♂1}$, $\mathbf{\Gamma}_{♀2} = \mathbf{\Gamma}_{♂2}$, $\mathbf{\Pi}_{♀1} = \mathbf{\Pi}_{♂1}$, $\mathbf{\Pi}_{♀2} = \mathbf{\Pi}_{♂2}$, and $\zeta_{♀} = \zeta_{♂}$

Table A2: Summary of the constraints applied to parameters in the SEMs.

A6 SOFTWARE

Models were implemented in JAGS 4.3.0 and R 4.0.0 (R Core Team 2020) with the use of the packages rjags 4-10 (Plummer 2016) and doMc 1.3.7 (Revolution Analytics and Weston 2015). For each model, we ran five MCMCs of 500,000 samples with a thin of 50, a burn-in of 300,000 and an adaptation phase of 1,000,000. As recommended by Lee and Song (2012) we choose parameter expanded normal priors or normal priors for all the regression parameters and inverse gamma priors for the precision parameters. The specification of prior distributions of the parameters differs among the models because invariant parameters among females and males share a single prior distribution, while not invariant parameters have independent prior specifications (Lee and Song 2012). Actual values of prior distributions can be found within the code to run the models on Gitlab:

https://gitlab.com/michebio/code_sem_reveals_determinants_of_fitness_in_cooperatively_breeding_bird

A7 RESULTS OMITTED IN THE MAIN TEXT

Variable	Threshold	Mean	lower 95% HPDI	upper 95% HPDI
number of offspring intra-group	α_1	9.9	9.46	10.33
number of offspring intra-group	α_2	22.13	21.21	23.09
number of offspring extra-group	α_1	12.35	11.86	12.82
number of offspring extra-group	α_2	21.7	20.8	22.58
number of offspring only one known parent	α_1	17.03	16.43	17.67
number of offspring only one known parent	α_2	24.46	23.28	25.64
group size	α_1	1.53	1.17	1.9
group size	α_2	11.53	11.07	11.99
number of helpers	α_1	8.64	8.23	9.05
number of helpers	α_2	19.01	18.29	19.72
insectXvegetation	α_1	-13.42	-14.02	-12.85
insectXvegetation	α_2	-4.98	-5.42	-4.55
insectXvegetation	α_3	1.92	1.51	2.36
insectXvegetation	α_4	7.89	7.41	8.35
territory size	α_1	-9.01	-9.44	-8.58
territory size	α_2	6.32	5.91	6.7
territory size	α_3	14.85	14.3	15.41

Table A3: Threshold estimates for the ordinal variables analyzed in the SEMs. For each variable, we report the threshold id, the mean estimate, the lower 95% high posterior density interval (HPDI), and the upper 95% HPDI. The number of threshold values equals the number of levels of the categorical variable minus one and varies between variables because each variable has a different number of levels. The number of offspring intra-group, extra-group and with only one known parent, the group size, and the number of helpers only had three levels of categories, while the insect per vegetation had five levels and the territory size had four levels.

Id	$L_{\nu=0.5}$	$\Delta_{L_{0.5}}$	Id	$L_{\nu=0.8}$	$\Delta_{L_{0.8}}$
$\boldsymbol{M6}$	21767.88	-	$\boldsymbol{M6}$	25883.84	-
$\boldsymbol{M3}$	21791.88	24.00	$\boldsymbol{M3}$	25918.76	34.92
$\boldsymbol{M1}$	21793.53	25.65	$\boldsymbol{M5}$	25920.47	36.63
$\boldsymbol{M4}$	21798.95	31.07	$\boldsymbol{M4}$	25921.55	37.71
$\boldsymbol{M5}$	21801.38	33.50	$\boldsymbol{M2}$	25999.46	115.62
$\boldsymbol{M2}$	21803.85	35.97	$\boldsymbol{M1}$	26007.18	123.34
$\boldsymbol{M8}$	22127.84	359.96	$\boldsymbol{M9}$	26553.72	669.88
$\boldsymbol{M9}$	22271.19	503.31	$\boldsymbol{M7}$	26570.66	686.82
$\boldsymbol{M7}$	22285.01	517.13	$\boldsymbol{M10}$	26583.66	699.82
$\boldsymbol{M10}$	22297.72	529.84	$\boldsymbol{M8}$	26953.67	1069.83

Table A4: Model ranking of the ten competing models based on the $L_{\nu=0.5}-$ and $L_{\nu=0.8}-$measure. The $\Delta_{L_{0.5}}$ and $\Delta_{L_{0.8}}$ represent the differece between each model and the top ranking model considering, respectively, the $L_{\nu=0.5}-$ and $L_{\nu=0.8}-$measure.

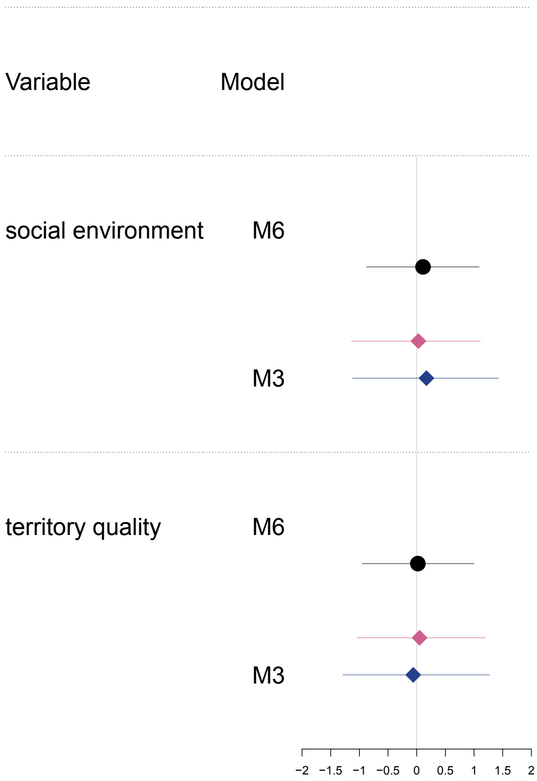

(a) Slopes of latent variables between-individual, $\mathbf{\Gamma}_2$

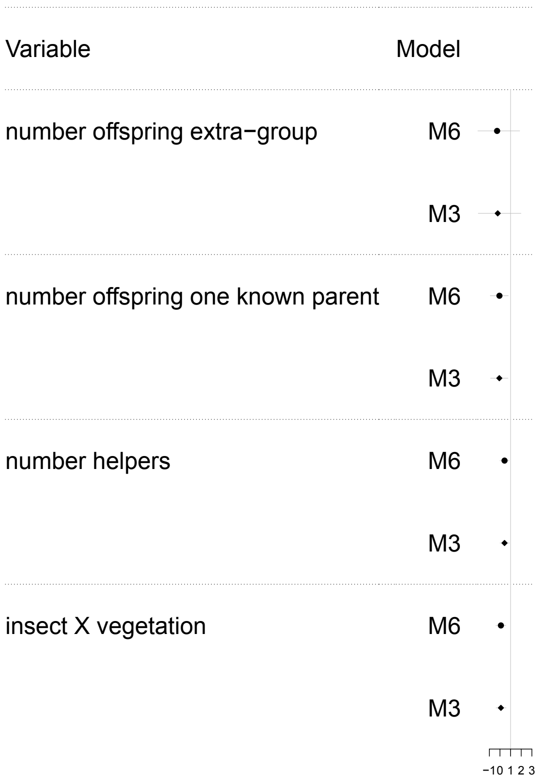

(b) Slopes of observed variables between-individual, $\mathbf{\Lambda}_{2\xi}$

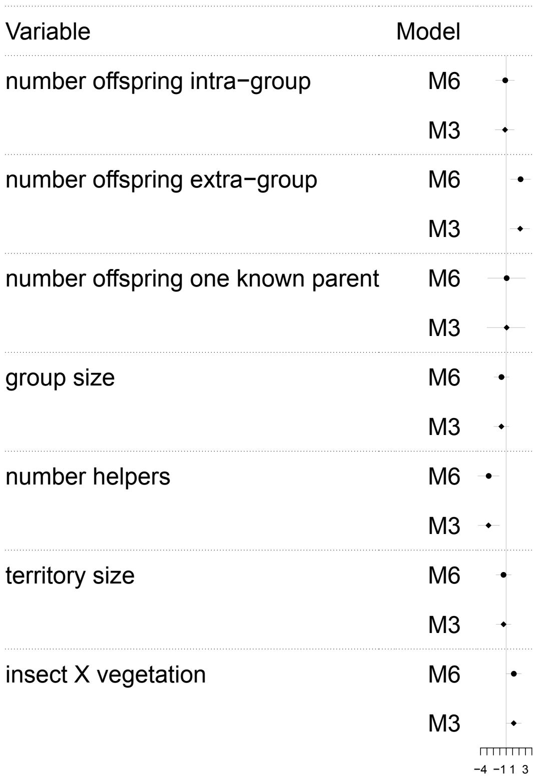

(c) Intercepts of observed variables within-individual, $\boldsymbol{\mu}$

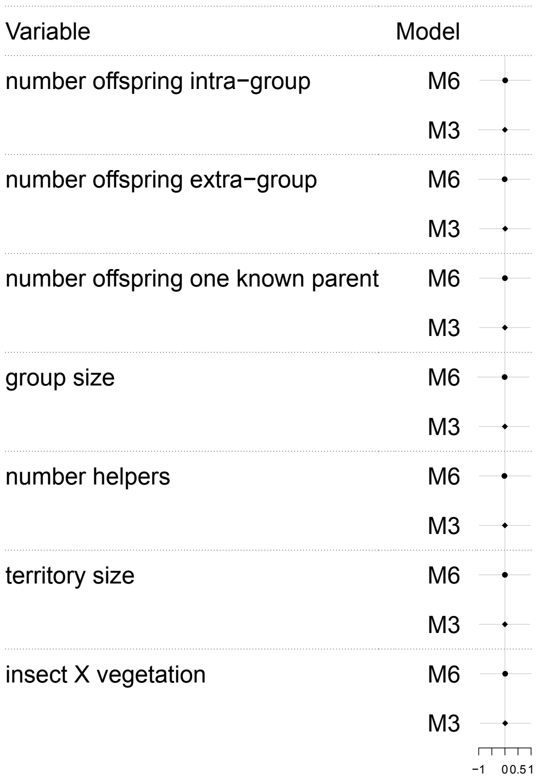

(d) Intercepts of observed variables between-pair, $\boldsymbol{\mu}_s$

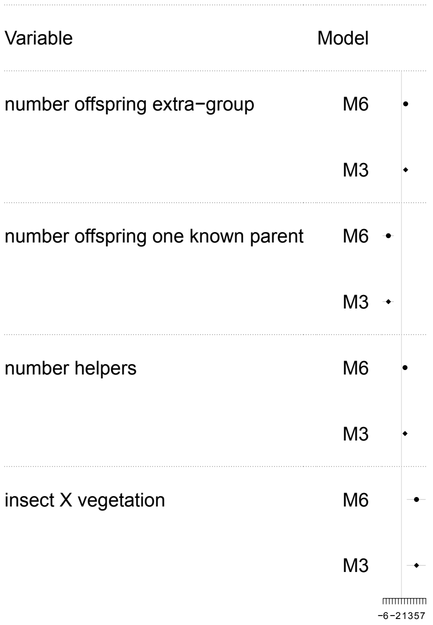

(e) Slopes of observed variables between-pair, $\mathbf{\Lambda}_s$

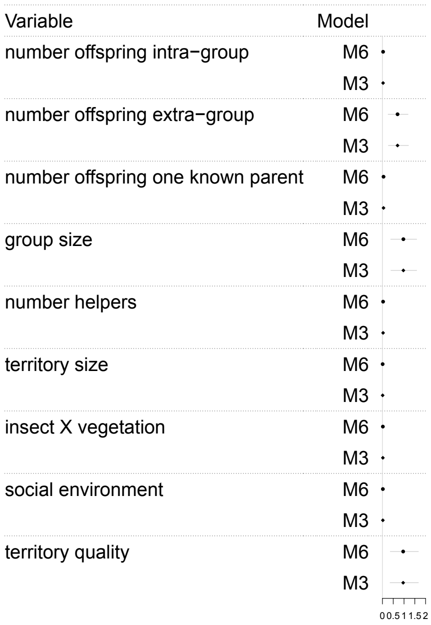

(f) Errors of observed variables between-pair, $\boldsymbol{\epsilon}_s$

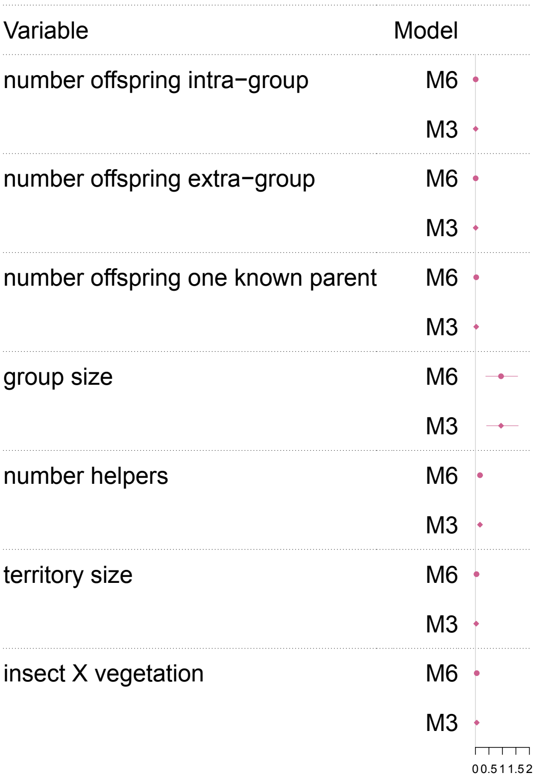

(g) Errors observed variables within-individual female, $\boldsymbol{\epsilon}_{♀1io}$

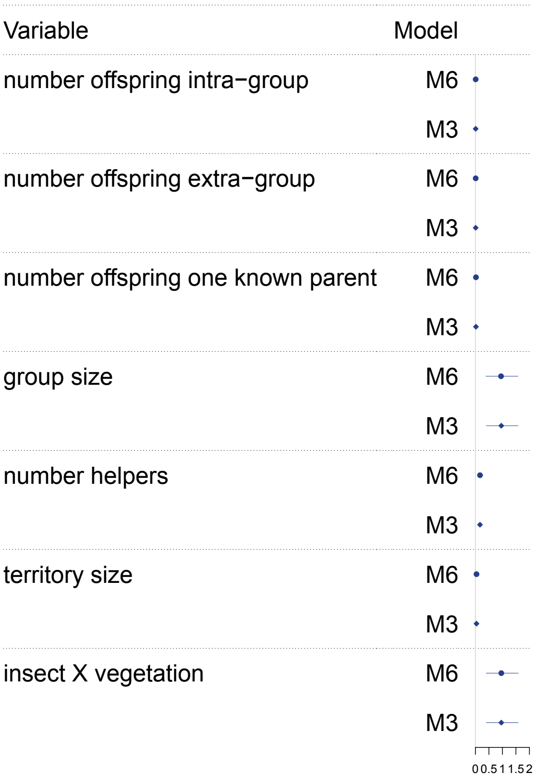

(h) Errors observed variables within-individual male, $\boldsymbol{\epsilon}_{♂1io}$

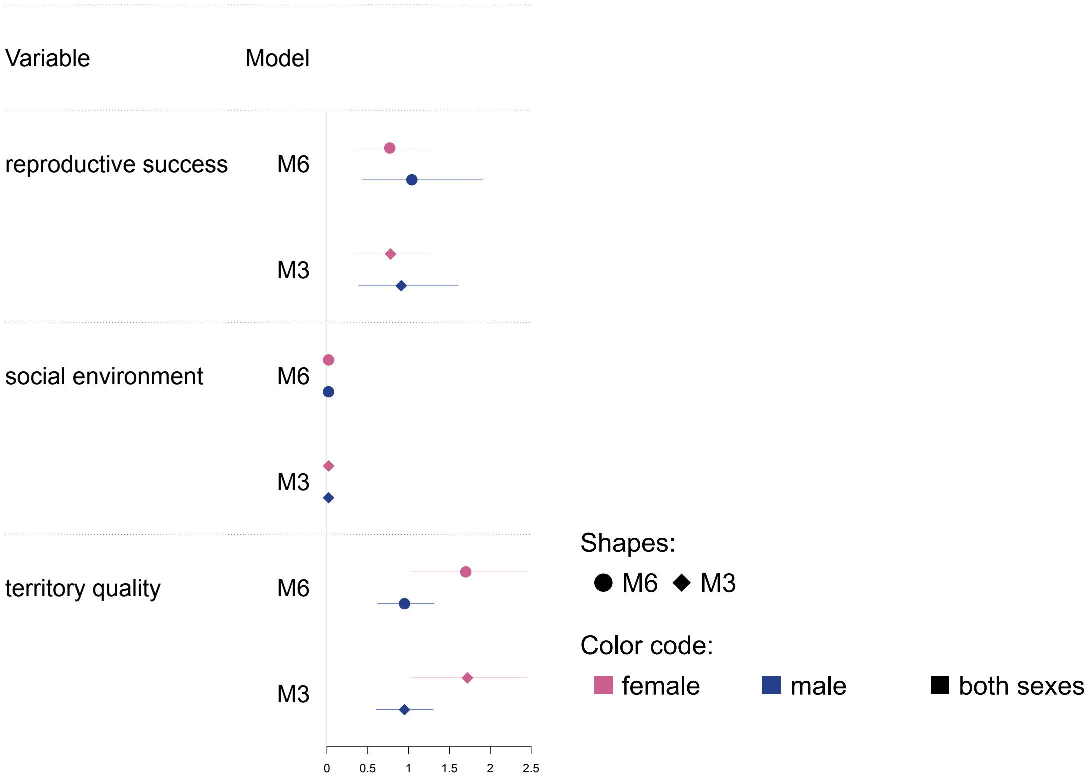

(i) Errors of the latent variable δ_{1io}

(j) Legend

Figure A3: Mean and 95%high-posterior density interval (HPDI) for the parameter estimates that are not reported in the main text for the best two models: $\boldsymbol{M6}$ (filled circles), and $\boldsymbol{M3}$ (diamonds). Pink symbols represent parameters of the sample of females, blue symbols of males, and black symbols of both sexes. Panel a represents the effect of the latent variables at the between-individual level, while Panel b represents the effect of the factor-loadings of the observed-variables between-individual. The values of $\mu = \mu_{♀} = \mu_{♂}$(Panel c), and μ_s (Panel d zYAHU xcfffffffffffffffffffffffffffffffffff represent the intercepts of the equations and should be interpreted as the value of the latent variable when the observed variables correspond to their mean. The production of extra-pair offspring highly drives variations in reproduction between social groups (Panel e). Variations in the number of helpers drive variations in the social environment between social groups (Panel e), while variations in the territory quality between social groups are mainly driven by insect availability rather than territory size (Panel e). Errors related to the estimation of the observed variables between social groups, of the observed variables within-individual female and male, and of the latent variables are reported in Panels f, g, h, and i respectively. Smaller mean error estimates indicate higher accuracy in the identification of that variable, smaller 95%HPDI indicate higher precision. Panel j shows the legend of the figure.

A8 INFO ABOUT DATA COLLECTION

Data on Seychelles warblers were collected on Cousin Islands ($29km^2$, $4°20'S$,$55°40'E$), Republic of Seychelles between 1995 and 2016. The population of Cousin island is constituted by circa 320 adult individuals and is at carrying capacity (Komdeur et al. 2016; Komdeur and Pels 2005). The population was enclosed into the island and migration/emigration to/from other islands was negligible (Komdeur et al. 2016). Monitoring was intense and almost all breeding attempts were recorded every year during the month of June and September, and ofter during January-March, which corresponds to the minor breeding season (Richardson et al. 2002).

Birds were caught using mist nets and marked with a metal ring from the British Trust for Ornithology and an individual color ring combination. At capture a small blood sample of circa $25\,\mu l$ was collected to determine the sex and assign parentage (Hadfield et al. 2006; Komdeur and Pels 2005). The age of the individuals was estimated based on a mixture of behavioral and morphological characteristics (Richardson and Burke 2003). Most birds were captured in their first year of age.

Thanks to molecular parentage assignment we were able to determine the identity of the parents (Edwards et al. 2016; Hadfield et al. 2006; Richardson et al. 2001; Sparks et al. 2020). The combination of molecular parentage assignment with behavioral observations and the identification of the individuals residing in a territory allowed us to identify if paternity was intra- or extra-group. However, for some offspring we were able to only assign the identity of one parent. The uncertainties in the determination of the mother or father arise because the population is highly inbred (i.e., close inbreeding occurred in circa 5% of matings, Richardson et al. 2004). Mothers were especially difficult to assign because the dominant breeding female and cobreeding helpers can be closely related (Richardson and Burke 2003).

Individuals live in groups and defend small territories with defined boundaries. The estimation of territory quality was derived from the territory size and food availability for each group. To calculate the surface of each territory (m^2), we draw the territory boundaries on a digital map in the ArcGIS 10.2 (ESRI) environment. To calculate the food abundance relative to the vegetation cover, we first scored the foliage coverage at 20 random points in each territory once a year. More specifically, at each random point, the foliage coverage of the tree species was scored visually at the following height bands: $0-$ to $0.75-$m, $0.75-$ to $2-$m and at two m intervals after that (Van de Crommenacker et al. 2011). Additionally, we calculated insect availability by counting the number of insects present under 50 leaves of the five most abundant tree species

present in 14 different regions across the island (Brouwer et al. 2009). These regions were chosen with similar exposition to wind-driven salt spray (Brouwer et al. 2009; Komdeur 1992), and insect counts taken in the center of each area were assumed to be representative for all the territories within that region (Komdeur 1992). Given the foliage cover per tree species with broad-leaves c_x and the mean monthly insect count i_x for each tree species x per unit leaf area we calculated the food availability as $\sum(c_x \, i_x)$ (dm^2; Komdeur 1992).

Each year, between June and September, all the residents in a territory older than six months were counted. The identity and social status of all these individuals was recorded through behavioral observations. When a dominant female started breeding, we performed at least 60 minutes of observations of the nest to determine which individuals visited the nest. If subordinate individuals were incubating the egg(s) and/or feeding the chick(s) they were assigned the status of helpers (Richardson et al. 2002). Some female helpers can lay an egg in the nest (co-breeding; Richardson et al. 2002), but their reproductive success was not analyzed here. There were 29 males that occupied two or more territories simultaneously. In those cases we took the average insectXvegetation, territory size, and we sum up the number of helpers and group size.

A9 COMPARISON WITH PREVIOUS STUDIES

Some of the results presented in the main text contradict previous studies on Seychelles warblers. We showed that territory quality did not impact the reproductive output of females and male breeders and that the number of helpers and group size had a positive effect on reproduction. However, Komdeur (1992) and Brouwer et al. (2009) showed a significant effect of territory quality and territory size on reproduction, respectively. Additionally, Brouwer et al. (2009) showed a positive effect of group size on reproduction only up to a group of five individuals. These discrepancies could be explained by either biological or methodological differences between the three studies. The biological differences could be linked to the different timelines of the three studies (see Table A4 which shows the little overlap between the timelines of the three studies). The studies also use different definitions of variables. For example, Komdeur (1992) and Brouwer et al. (2009) analyzed the number of fledglings that survived to at least three months of age as a proxy for reproduction, while here we analyzed the number of offspring that reached at least 12 months/one year of age. The definition of territory quality also differs: Komdeur (1992) defines territory quality with three categories (low, medium, high quality); Brouwer et al. (2009) calculated the territory size over the number of individuals in the territory; and here, we analyzed the simultaneous effects of food availability and territory size within a latent variable. The main methodological differences are the implementation of generalized linear models (GLM) by Komdeur (1992) and generalized mixed models (GLMM) by Brouwer et al. (2009) versus SEM in this study. There are also other technical differences worth mentioning. The GLM/GLMM in a frequentist framework analyzed the reproductive output of the breeding pair and did not impute missing data. In contrast, with Bayesian SEM, we analyzed the reproductive performance of females and males and imputed missing data points. To tease apart if biological or methodological differences could explain the discrepancies in the conclusions, we applied the statistical models presented in Brouwer et al. (2009) and Komdeur (1992) to our dataset collected between 1995 and 2016. We ran the GLMs/GLMMs in R (R Core Team 2020) with the glmmTMB package (v. 1.1.2 Bolker 2016) and a log link for count data. Competing models were ranked from the lowest to the highest Akaike information criterion (AIC) values, where a lower AIC indicates a better model fit to the data (Akaike 1987). We present the results of these additional analyses in the following subsections. We predict that the discrepancy between our results with previous studies will be due to:

- biological differences if the implementation of GLM/GLMM on the long-term dataset pro-

vides comparable conclusions with parts of the SEM analysis; or

- methodological differences if results of the GLM/GLMM on the long-term dataset provide comparable conclusions with previous research.

We analyzed both the number of fledglings produced and the number of offspring that reached 12+ months of age (both calculated from the pedigree) with separate GLMs/GLMMs. We speculate that potential discrepancies between the results of the GLM/GLMM on the number of fledglings versus the number of offsprings of 12+ months of age could indicate biological differences in the effect of territory quality and the social environment in the survival of offsprings up to 12+ months of age.

Please note that in the main text, we also refer to a study by Brouwer et al. (2006) who focused on survival. We do not present a formal comparison here as our study focuses on reproduction only.

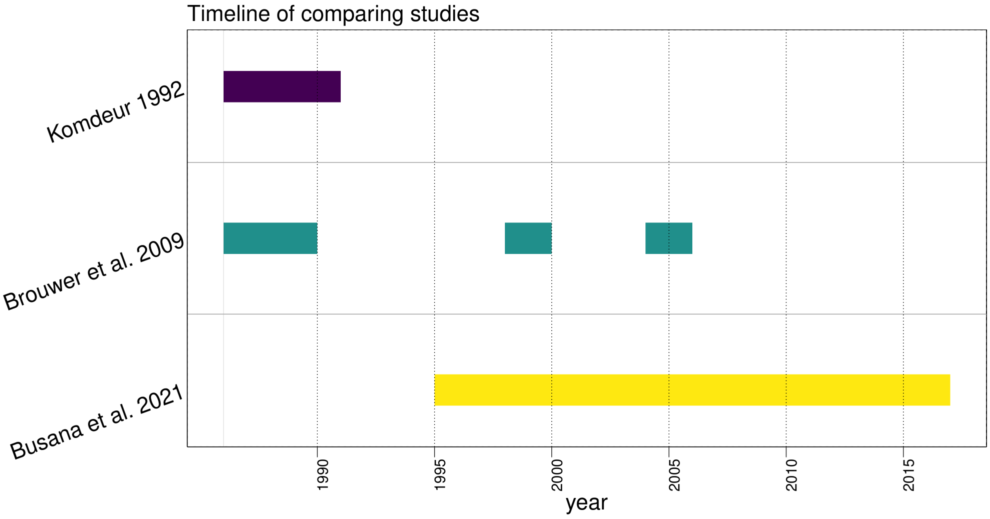

Figure A4: Years of data analyzed by Komdeur (1992, years 1986-1990, purple), Brouwer et al. (2009, yeas 1986-1989 + 1998-1999 + 2004-2005, blue) and this study (yellow, 1995-2016).

A9.1 Comparison with Komdeur 1992

Komdeur (1992) showed that yearly reproductive success (the number of fledglings of at least three months of age produced) increased with territory quality and when helpers were present to aid the breeding pair (we copy here the original Table 1 from the paper, now Fig. A5). Following the results from Komdeur (1992), we applied a GLM to our dataset. We ran separate models for the number of fledglings and the number of offsprings of 12+ months. The explanatory variables considered were territory quality (low/medium/high quality where low corresponds to values of territory quality less or equal to the 33rd quantile, medium to values above the 33rd quantile and less or equal to the 66th quantile, and high to values above the 66th quantile), helper(s) presence, and their interaction. Results are presented in Table A5-A6). The model with the lowest AIC value was a model with a single predictive variable, helper presence, both for the number of fledglings and the number of offsprings of 12+ months (Table A5-a and -b). Therefore with the data from 1995-2016 and according to GLMs, there was no effect of territory quality on reproductive output. The effect of helpers' presence was positive both when analyzing the number of fledglings and the number of offspring of 12+ months of age (see Table A6-a1 and -b1, minimal models). For consistency and to easily compare the results with Table 1 (now Fig. A5) from Komdeur (1992) we also report results of the full model with the interaction between helper(s) presence and territory quality (see Table A6-a2 and -b2, full models). The parameter estimates of the two full models indicate that territory quality did not significantly affect the reproductive output of the breeding pair. Overall, these results meet our first prediction and

show that the discrepancy between the SEM analysis and Komdeur (1992) is due to biological differences.

TABLE 1 Effects of territory quality on the Seychelles warbler on Cousin Island (1986–1990)

	Territory quality†			
	Low	Medium	High	Statistic‡
Yearly reproductive success per unaided pair§	0.19 ± 0.12 (286)	0.51 ± 0.19 (38)	0.85 ± 0.21 (28)	$H = 101.04$[illegible]
Yearly reproductive success per group (V)§ (pair plus helpers)	0.22 ± 0.13 (365)	0.85 ± 0.21 (60)	1.27 ± 0.36 (55)	$H = 161.35$[illegible]
Mean group size	2.4 ± 0.21 (365)	2.9 ± 0.91 (60)	3.7 ± 0.93 (55)	$H = 110.12$[illegible]
First-year survival (s)‖	0.30 (103)	0.67 (23)	0.86 (22)	$X^2 = 28.85$*
Annual adult survival (s)‖	0.76 (156)	0.88 (64)	0.91 (48)	$X^2 = 7.82$**
Mean adult life expectancy (z)	2.7	5.4	7.4	

The table shows the effect of territory quality on breeding success and mean group size (±standard deviation; numbers in parentheses are territory years), first-year survival (the probability of surviving to age of one year) and annual adult survival (probability of surviving to the next year, starting at age of one year) (numbers in parentheses are bird years based on colour-ringed individuals), and mean adult life expectancy (the length of time after which 50% of the population alive at one year will have died). A pair consists of one male and one female. There was no emigration from the island, so birds that disappeared could be reliably scored as dead. Seychelles warbler pairs occupying high-quality territories fledged significantly more young per year than pairs on lower-quality territories. First year survival and annual adult survival increased significantly with territory quality. Mean adult life expectancy was therefore also significantly correlated with territory quality.

Figure A5: Table 1 copied with permission from Komdeur (1992)

(a) number of fledglings surviving to 3+ months of age			
Predictors	df	AIC	Δ
helper presence	2	2766.53	0
territory quality and helper presence (without interaction)	4	2768.70	2.17
interaction between territory quality and helper presence	6	2771.98	5.45
null model	1	2796.04	29.51
territory quality	3	2798.00	31.47
**(b) number of offsprings surviving to 12+ months of age**			
Predictors	df	AIC	Δ
helper presence	2	3951.42	0.00
null model	1	3954.51	3.10
territory quality and helper presence (without interaction)	4	3955.34	3.92
territory quality	3	3958.41	6.99
interaction between territory quality and helper presence	6	3959.14	7.72

Table A5: Generalized linear models (GLMs) rankings when replicating the analyses from Komdeur (1992). The dependent variables considered were the number of fledglings (3+ months of age) and offsprings surviving to at least 12+ months of age. The preferred model with the lowest Akaike information criterion (AIC) included only helper presence as an explanatory variable in both cases. Column df represents the degrees of freedom of each model. The values of Δ are the differences in AIC values between the preferred model and each model.

Type	Predictors	(a1) number of fledglings surviving to 3+ months of age			(b1) number of offsprings surviving to 12+ months of age		
**MINIMAL MODELS**							
**Type**	**Predictors**	**Log-Mean**	**std. Error**	**CI**	**Log-Mean**	**std. Error**	**CI**
Intercept	no helper	**-1.03**	**0.05**	**-1.12 – -0.94**	**0.24**	**0.02**	**0.20 – 0.29**
Contrast	helper(s)	**0.48**	**0.08**	**0.32 – 0.64**	**0.11**	**0.05**	**0.02 – 0.21**
	Observations	1665			1665		
**FULL MODELS**							
		**(a2) number of fledglings surviving to 3+ months of age**			**(b2) number of offsprings surviving to 12+ months of age**		
**Type**	**Predictors**	**Log-Mean**	**std. Error**	**CI**	**Log-Mean**	**std. Error**	**CI**
Intercept	no helper in low TQ	**-1.10**	**0.08**	**-1.27 – -0.94**	**0.24**	**0.04**	**0.15 – 0.32**
Contrast	no helper in medium TQ	0.13	0.11	-0.10 – 0.35	0.01	0.06	-0.11 – 0.13
Contrast	no helper in high TQ	0.10	0.12	-0.13 – 0.32	0.00	0.06	-0.11 – 0.12
Contrast	helper(s) in low TQ	**0.48**	**0.15**	**0.19 – 0.77**	0.10	0.09	-0.07 – 0.27
Contrast	helper(s) in medium TQ	**0.51**	**0.15**	**0.21 – 0.80**	0.10	0.09	-0.07 – 0.28
Contrast	helper(s) in high TQ	**0.65**	**0.14**	**0.38 – 0.91**	0.15	0.08	-0.02 – 0.31
	Observations	1665			1665		

Table A6: Intercepts and contrasts for Poisson models (log link) of the yearly number of offspring that survived to at least three months of age (fledglings, left-hand-side) and at least 12 months of age (right-hand side). Parameters are untransformed (i.e. on the log-scale or log-mean). Explanatory variables considered are the absence or presence of helpers (respectively helpers(s) and no helper) and their interaction with territory quality (respectively low, medium, high TQ). We report the parameter estimates for the minimal models on the top of the table and on the bottom of the table the full models. The column Type indicates if the variable is an intercept or the contrast between a variable and the intercept. The column std. Error represents the standard error, while CI represents the 95% confidence intervals of each parameter. When the confidence intervals did not overlap zero (i.e. there was a significant effect of the corresponding predictor), we highlighted the predictor with the bold font.

A9.2 Comparison with Brouwer et al. 2009

Brouwer et al. (2009) showed that the number of fledglings produced by the breeding pair increased with the relative territory size per bird (or per capita territory size, equal to the ratio between territory size and group size) and with group size up to a group size of five, but decreased afterward (the maximum group size observed was seven). To facilitate comparisons, we copy here Table 2 from Brouwer et al. 2009, now Fig. A6. The GLMMs included year and territory ID (a unique identifier for each territory) as random effects. We replicated the GLMMs for the number of fledglings and offsprings of 12+ months of age with data from 1995-2016. We considered per capita territory size (territory/bird), group size, and group size squared as explanatory variables. Model rankings based on the AIC values showed that the preferred models differ when considering the number of fledglings (Table A7-a) and the number of offspring of 12+ months of age (Table A7-b). When analyzing the number of fledglings produced, the GLMM with relative territory size per bird, group size and group size squared was the preferred model (Table A7-a). Therefore the full model was the preferred model, and the results were similar to those of Brouwer et al. (2009). We observed a positive effect of the relative territory size per bird and a quadratic effect of group size with an initial increase and a decline when there were two or more additional individuals in a group on the number of fledglings produced (Table A8-a1). The GLMM results differ when analysing the number of offspring of 12+ months of age (Table A7-b and A8-b1 and -b2). The minimal model, including only group size, had a lower AIC value and was the preferred model (Table A7-b). We observed a positive linear effect of group size on the reproductive output of the breeding pair (Table A8-b2). For exhaustivity, we also report the full model at the top right of Table A8-b1 confirming that only the parameter estimates for group size had a significant positive effect on the number of offspring of 12+months of age. The agreement between the results of the GLMM and SEM on the number of offspring of 12+ months of age indicates that different conclusions with Brouwer et al. (2009) were due to biological differences. Since the analysis of the number of fledglings with GLMMs produced similar results with Brouwer et al. (2009) we speculate that the biological differences might be linked with the differential effect of group size and relative territory size on the survival of fledglings to adulthood. Our analyses are preliminary, and further investigations should be conducted to tackle why these differences might occur.

Table 2. Results from analyses examining annual reproductive output per territory (no. fledglings) of Seychelles Warblers on Cousin Island for 8 years between 1986 and 2006 ($N = 902$ territories).

Parameter	$B \pm$ SE	χ^2	df	P
Final model				
Intercept	−2.91 ± 0.38		1	
Territory size/bird	3.24 ± 0.81	14.0	1	<0.001
Group size	1.12 ± 0.20	27.4	1	<0.001
Group size^2	−0.09 ± 0.03	11.7	1	<0.001
Random effects				
σ^2_{year}	0.16 ± 0.15			
$\sigma^2_{territory}$	0.01 ± 0.01			
Rejected				
Population density	0.14 ± 0.20	0.50	1	0.48
Territory quality	0.002 ± 0.01	0.02	1	0.89
Total rainfall	0.001 ± 0.001	0.46	1	0.50
Breeding season rainfall	0.001 ± 0.001	0.10	1	0.75

Figure A6: Table 2 copied with permission from Brouwer et al. (2009)

(a) number of fledglings surviving to 3+ months of age			
Predictors	df	AIC	Δ
(territory size / bird) + group size + group size^2	6	3866.74	0
group size + group size^2	5	3872.48	5.74
(territory size / bird) + group size	5	3877.15	10.42
group size	4	3878.45	11.71
(territory size / bird)	4	3949.96	83.22
null model	3	3975.02	108.29
**(b) number of offsprings surviving to 12+ months of age**			
Predictors	df	AIC	Δ
group size	4	5401.28	0
group size + group size^2	5	5401.47	0.19
(territory size / bird) + group size	5	5403.27	1.99
(territory size / bird) + group size + group size^2	6	5403.30	2.02
(territory size / bird)	4	5412.76	11.48
null model	3	5417.98	16.70

Table A7: Generalized linear mixed models (GLMMs) rankings when replicating the analyses from Brouwer et al. (2009). The dependent variables considered were the number of fledglings and the number of offspring surviving to at least 12+ months of age. When analyzing the number of fledglings the preferred model with the lowest Akaike information criterion (AIC) was the full model including per capita territory size (territory size/bird), group size, and its square. When analyzing the number of offspring of 12+ months of age the preferred model based on its AIC value contained only group size. The column df represents the degrees of freedom of each model. The values of Δ are the differences in AIC values between the preferred model and each model.

FULL MODELS	(a1) number of fledglings surviving to 3+ months of age			(b1) number of offsprings surviving to 12+ months of age		
**Predictors**	**Log-Mean**	**std. Error**	**CI**	**Log-Mean**	**std. Error**	**CI**
Intercept	-0.30	0.24	-0.78 – 0.17	0.46	0.13	**0.21 – 0.71**
(territory size/ bird)	0.14	0.05	**0.04 – 0.24**	0.01	0.03	-0.04 – 0.06
group size	1.07	0.21	**0.67 – 1.48**	0.24	0.12	**0.01 – 0.48**
group size^2	-0.25	0.07	**-0.39 – -0.11**	-0.06	0.04	-0.14 – 0.02
**Random Effects**						
σ^2	1.31			0.56		
τ_{00} year	0.31			0.01		
τ_{00} territory	0.08			0		
N year	21			21		
N territory	178			178		
Observations	2258			2258		
Marginal R^2	0.068			0.012		

MINIMAL MODELS	(a2) number of fledglings surviving to 3+ months of age			(b2) number of offsprings surviving to 12+ months of age		
**Predictors**	**Log-Mean**	**std. Error**	**CI**	**Log-Mean**	**std. Error**	**CI**
Intercept		Not applicable		0.28	0.02	**0.24 – 0.33**
group size		Not applicable		0.08	0.02	**0.04 – 0.11**
**Random Effects**						
σ^2		Not applicable		0.56		
τ_{00} year		Not applicable		0.01		
τ_{00} territory		Not applicable		0.00		
N year		Not applicable		21		
N territory		Not applicable		178		
Observations		Not applicable		2258		
Marginal R^2		Not applicable		0.011		

Table A8: Intercepts and slopes for Poisson models of the yearly number of offspring that survived to at least 3 months of age (fledglings, left-hand-side) and 12+ months/one+ year of age (right-hand side) as a function of per capita territory size (territory size/bird), group size and the squared of group size. Parameters are untransformed (i.e. in the log-scale or log-mean). On the top of the table we report the parameter estimates for the full models and on the bottom of the table the minimal model. When analyzing the number of fledglings produced the preferred model was the full model. When considering the number of offspring of 12+ months of age the preferred model was the minimal model. The column std. Error represents the standard error, while CI represents the 95% confidence intervals of each parameter. When the confidence intervals did not overlap zero (i.e. there was a significant effect of the corresponding predictor) we highlighted the predictor with the bold font.

BIBLIOGRAPHY

Akaike H. 1987. Factor analysis and aic, In: Selected papers of hirotugu akaike, Springer, pp. 371–386.

Bolker B. 2016. Getting started with the glmmtmb package. Vienna, Austria: R Foundation for Statistical Computing. software. .

Brouwer L, Richardson DS, Eikenaar C, Komdeur J. 2006. The role of group size and environmental factors on survival in a cooperatively breeding tropical passerine. J. Anim. Ecol. 75:1321–1329.

Brouwer L, Tinbergen JM, Both C, Bristol R, Richardson DS, Komdeur J. 2009. Experimental evidence for density-dependent reproduction in a cooperatively breeding passerine. Ecology. 90:729–741.

Cubaynes S, Doutrelant C, Grégoire A, Perret P, Faivre B, Gimenez O. 2012. Testing hypotheses in evolutionary ecology with imperfect detection: capture–recapture struct. eqn. modeling. Ecology. 93:248–255.

Edwards HA, Dugdale HL, Richardson DS, Komdeur J, Burke T. 2016. Exploration is dependent on reproductive state, not social state, in a cooperatively breeding bird. Behav. Ecol. 27:1889–1896.

Gelman A, Carlin JB, Stern HS, Dunson DB, Vehtari A, Rubin DB. 2014. Bayesian data analysis, vol. 2, 2nd ed., Boca Raton, FL: CRC press, pp. 419–420.

Hadfield JD, Richardson DS, Burke T. 2006. Towards unbiased parentage assignment: Combining genetic, behavioural and spatial data in a Bayesian framework. Mol. Ecol. 15:3715–3730.

Komdeur J. 1992. Importance of habitat saturation and territory quality for evolution of cooperative breeding in the Seychelles warbler. Nature. 356:133–135.

Komdeur J, Burke T, Dugdale HL, Richardson DS. 2016. Seychelles warblers: Complexities of the helping paradox, In: Koenig WD, Dickinson JL, editors, Cooperative Breeding in Vertebrates: Studies of Ecology, Evolution, and Behavior, Cambridge University Press, p. 197–216.

Komdeur J, Pels MD. 2005. Rescue of the Seychelles warbler on Cousin Island, Seychelles: The role of habitat restoration. Biol. Conserv. 124:15–26.

Lee SY. 2007. Struct. Eqn. Modeling: A Bayesian approach, vol. 711, John Wiley & Sons, pp. 139–142.

Lee SY, Song XY. 2012. Basic and advanced Bayesian Struct. Eqn. Modeling, Wiley-Blackwell.

Plummer M. 2016. rjags: Bayesian Graphical Models using MCMC. R package version 4-6.

R Core Team. 2020. R: A Language and Environment for Statistical Computing, R Foundation for Statistical Computing, Vienna, Austria.

Revolution Analytics, Weston S. 2015. doMC: Foreach Parallel Adaptor for 'parallel'. R package version 1.3.4.

Richardson DS, Burke T. 2003. Sex-specific associative learning cues and inclusive fitness benefits in the Seychelles warbler. J. Evol. Biol. 16:854–861.

Richardson DS, Burke T, Komdeur J. 2002. Direct benefits and the Evolution of Female-biased Cooperative Breeding in Seychelles Warblers. Evolution. 56:2313–2321.

Richardson DS, Jury FL, Blaakmeer K, Komdeur J, Burke T. 2001. Parentage assignment and extra-group paternity in a cooperative breeder: The Seychelles warbler (*Acrocephalus sechellensis*). Mol. Ecol. 10:2263–2273.

Richardson DS, Komdeur J, Burke T. 2004. Inbreeding in the seychelles warbler: environment-dependent maternal effects. Evolution. 58:2037–2048.

Song XY, Lu ZH, Hser YI, Lee SY. 2011. A bayesian approach for analyzing longitudinal structural equation models. Struct. Eqn. Modeling. 18:183–194.

Sparks AM, Spurgin LG, van der Velde M, Fairfield EA, Komdeur J, Burke T, Richardson DS, Dugdale H. 2020. Telomere heritability and parental age at conception effects in a wild avian population. Mol. Ecol. .

Thanoon TY, Adnan R. 2015. Bayesian analysis of multiple group non-linear structural equation models with ordered categorical and dichotomous variables: A survey. Research Journal of Mathematical and Statistical Sciences, ISSN. 2320:6047.

Van de Crommenacker J, Komdeur J, Burke T, Richardson DS. 2011. Spatio-temporal variation in territory quality and oxidative status: A natural experiment in the Seychelles warbler (*Acrocephalus sechellensis*). J. Anim. Ecol. 80:668–680.
